# Isonicotinamide-Based Compounds: From Cocrystal to Polymer

**DOI:** 10.3390/molecules24224169

**Published:** 2019-11-17

**Authors:** Francisco Sánchez-Férez, Daniel Ejarque, Teresa Calvet, Mercè Font-Bardia, Josefina Pons

**Affiliations:** 1 Departament de Química, Universitat Autònoma de Barcelona, 08193 Barcelona, Spain; francisco.sanchez.ferez@uab.cat (F.S.-F.); daniel.ejarque@e-campus.uab.cat (D.E.); 2 Cristal·lografia, Mineralogia i Dipòsits Minerals, Universitat de Barcelona, 08028 Barcelona, Spain; mtcalvet@ub.edu; 3 Unitat de Difracció de Raig-X, Centres Científics i Tecnològics de la Universitat de Barcelona (CCiYUB), Universitat de Barcelona, 08028 Barcelona, Spain; mercef@ccit.ub.edu

**Keywords:** cocrystal, Cu(II) compounds, isonicotinamide, amide–amide pattern, coordination polymer

## Abstract

The reaction between [Cu(μ-OAc)(μ-Pip)(MeOH)]_2_ (**1**) (OAc = acetate; Pip = 1,3-benzodioxole-5-carboxylate) and isonicotinamide (Isn) in MeOH as solvent yielded two mixture pairs of three compounds: {(HPip)_2_(Isn) (**2**), [Cu(Pip)_2_(Isn)_2_] (**3**)} and {(**3**), {[Cu_3_(Pip)_2_(OAc)_2_(μ-Isn)_2_(Isn)_2_(μ-OCH_3_)_2_(MeOH)_2_]·2MeOH}_n_ (**4**)}. Modifying the reaction conditions (t, T, molar ratio), **2** and **3** have been successfully isolated, whereas **3** and **4** had to be mechanically separated. The recrystallization of **3** in pentanol yielded single crystals of compound [Cu(Pip)_2_(Isn)_2_]·C_5_H_11_OH (**3a**). The X-ray crystal structure of **2**, **3a**, and **4** has been elucidated showing a cocrystal, a monomer, and an unusual coordination polymer, respectively. The Pip ligand exhibited a chelate (**3a**) or a monodentate (**4**) coordination mode, but the Isonicotinamide (Isn) ligand is the one that promoted the arrangement of different structures and also mainly directs the formation of the 2D and 3D supramolecular assemblies. All the structures have been analyzed by Hirshfeld surface. In addition, the energy frameworks and lattice energy values of **2** and **3a** have been calculated.

## 1. Introduction

The design of new organic and inorganic materials linked by intermolecular forces is a topic of current interest as models for biological systems and in materials science [1,2]. In this sense, hydrogen bonds are the main directing motif and are raised as one of the strongest and most used tools for crystal engineering [3,4]. Their large electrostatic components facilitate its ordering as first suggested by Etter’s rules—the best hydrogen bond donor will associate with the best hydrogen bond acceptor [5] and subsequently superseded by supramolecular suitable directing motifs (synthons), which imply functional groups [6,7]. Synthons are the backbone to design novel structures based on hierarchical, and therefore predictable, associations of the parts involved in them.

Above all, carboxylic acids can form strong hydrogen bonds through their acidic moiety, which in combination with *N*-containing groups (amines, amides, or pyridines) are replaced by the corresponding N-H···O interactions. The strength of this association is governed by the acidic and basic character of both parties involved in it [8]. This has led to the formation of single and multi-component crystals made of organic molecules named cocrystals [9]. The importance of cocrystals lies in the possibility to modify the physical properties of their components (melting point, solubility, or conductivity, among others) while keeping their chemical properties unaltered. This goal is achieved by the rearrangement of the intermolecular interactions promoted by the components and, thus, the formation of a new crystal structure [10,11]. It should be noted that during the formation of these assemblies, and in presence of a metal ion, the Isonicotinamide (Isn) units can interact either with the acidic moiety or with the metal center, wherefore a competition with the final complex formation occurs [12,13].

Among all the possible coformers, Isn stands out from the rest for its potential acceptor ability and therefore, it is considered a valuable component in the formation of cocrystals [14]. The Isn synthon itself promotes the amide···amide hydrogen bond interaction, ordering in a linear fashion but pointing in opposite direction. The pK_a_ rule allows to ascertain if the combination of the selected acid and base will form a salt or a cocrystal. If ΔpK_a_ < 0, the carboxylic acid···pyridine (O–H···N) interaction will be neutral [15,16]. For this reason, 1,3-benzodioxole-5-carboxylic acid (HPip, pK_a_ = 4.35) has raised as a prospective candidate, owing to the fact that it obeys this rule and, thus, should form cocrystals with Isn (pK_b_ = 10.87).

Besides, Isn formed numerous coordination complexes promoting their assembly in supramolecular structures mainly directed by the amide···amide pattern, even in competitive interactions with additional directing motifs [17,18,19,20]. In turn, carboxylates have a large versatility of coordination modes and can be used as a building block including Isn [21,22]. Among them, Cu(II) nodes possess high affinity toward oxygen donor atoms, especially belonging to carboxylate motifs and formed thousands of complexes. For this reason, metal carboxylates have been raised as one of the major interesting research items concerning their structural versatility and labile nature [23,24].

**Scheme 1:**
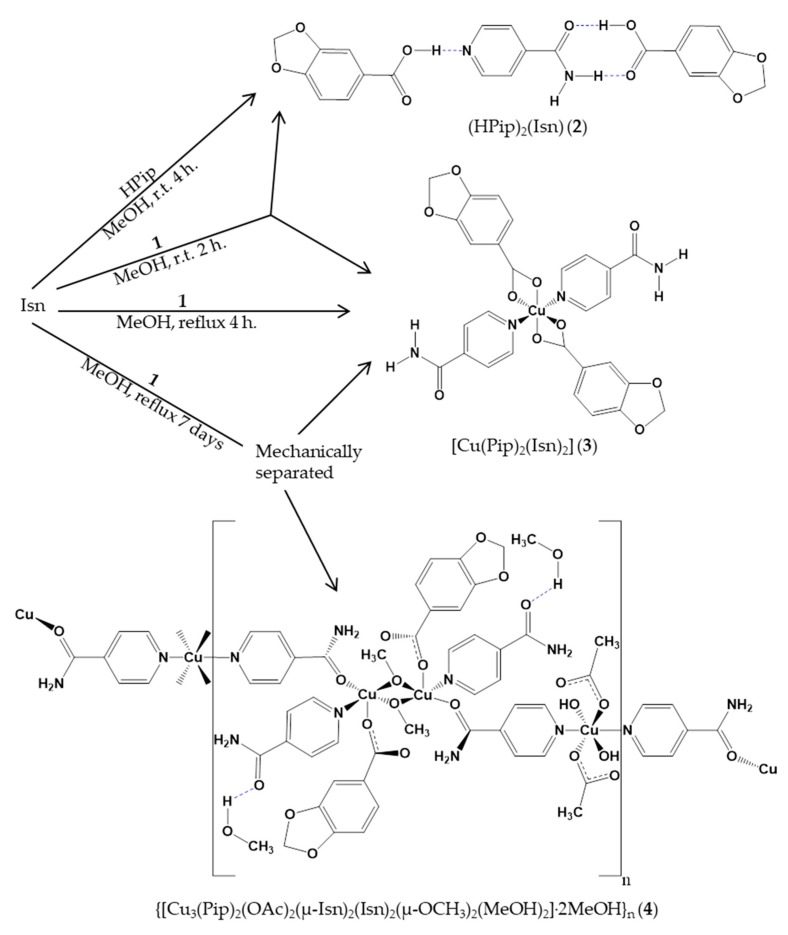
Outline of the synthesis of compounds **2**–**4**.

Previously, our group evinced the solvent-dependent formation of five Cu(II) compounds—one monomer and four dimers—resulting from the reaction between [Cu(μ-OAc)(μ-Pip)(MeOH)]_2_ (**1**) and Isn in different solvents. It should be noted that, in all of them, the Pip ligands were displaced, and only acetate ligands remained coordinated [25]. As a continuation, in this contribution we have assayed the reactions between **1** and Isn in order to synthesize compounds containing both Pip and Isn ligands in their structures. With this aim, we have modified the reaction conditions (reaction time, temperature, concentration, etc.). This made it possible to isolate three compounds: one cocrystal ((HPip)_2_(Isn), **2**), one Cu(II) monomer ([Cu(Pip)_2_(Isn)_2_], **3**), and a Cu(II) coordination polymer ({[Cu_3_(Pip)_2_(OAc)_2_(μ-Isn)_2_(Isn)_2_(μ-OCH_3_)_2_(MeOH)_2_]·2MeOH}_n_, **4**) (Scheme 1). Compound **3** was recrystallized in pentanol yielding [Cu(Pip)_2_(Isn)_2_]·C_5_H_11_OH (**3a**). Hirshfeld surfaces analysis as well as Energy frameworks and Lattice energy calculations facilitated the better understanding of the Isn role in these systems.

## 2. Results and Discussion

### 2.1. Synthesis and Characterization of Cocrystal **2**

The reaction of **1** [26,27] with Isn in MeOH as solvent at room temperature (r.t.) for 2h yielded a mixture of compounds (HPip)_2_(Isn) (**2**) and [Cu(Pip)_2_(Isn)_2_] (**3**). Otherwise, when the reaction was performed under reflux, only compound **3** was obtained. Following this, compound **2** was isolated by crystallizing a mixture of HPip:Isn (2:1 molar ratio) in MeOH at r.t. In this work, the reactions were performed without adding acetic acid, and the experimental conditions (t, T or concentration) have been modified in order to incorporate the Pip units in the structure of the complexes [28].

Compound **2** was characterized by EA, FTIR-ATR, ^1^H- and ^13^C{^1^H}-NMR spectroscopies, and single crystal X-ray diffraction method. The FTIR-ATR and ^1^H- and ^13^C{^1^H}-NMR spectra in dmso-*d*_6_ solution displays the characteristic bands of the two molecules (Figures S1–S3). In the FTIR-ATR spectrum (Figure S1a), the ν(N-H) bands appear at 3321 and 3140 cm^−1^ and are shifted with respect to the free Isn (3359 and 3179 cm^−1^, respectively) (Figure S1b). There is a broad signal at 1680 cm^−1^, assigned to the ν(C=O)_Isn_ and ν(COOH)_HPip_, which is also shifted compared to the HPip- (Figure S1c) and Isn-free molecules (1667 and 1655 cm^−1^, respectively).

Additional peaks can be identified as δ(O=CN), δ_ip_(C-H) and δ_oop_(C-H) [29]. The ^1^H- and ^13^C{^1^H}-NMR spectra show no displacement of the bands respect to the free molecules. The ^1^H-NMR spectrum confirms the 2:1 proportion of the coformers (Figures S2 and S3).

Therefore, the EA, FTIR-ATR, and ^1^H- and ^13^C{^1^H}-NMR data are in agreement with the structures determined by single crystal X-ray diffraction method. The ΔpK_a_ (pK_a_ conjugate acid of pyridine—pK_a_ carboxylic acid)) between the parties which form the association is a determining factor for the formation of a salt or a cocrystal. If ΔpKa < 0 or ΔpKa > 3.75, a cocrystal or a salt will be formed, respectively. If 0 < ΔpK_a_ < 3.75, there is an intermediate interaction, and either cocrystal or salt can be found [16].

### 2.2. Crystal Structure of Compound **2**

Compound **2** crystallizes in the triclinic P$\bar{1}$ space group. Its crystal structure can be described as the sum of the interactions between its functional groups. The position and nature of each functionality generates the particular interactions, which dictate the crystal arrangement [4,29]. The association of HPip and Isn leads to the formation of a cocrystal [30], in which there is not any homomeric interaction and therefore, the assembly has grown mixing molecules. In this kind of multi-component solids, salts or cocrystals can be formed depending on where the proton is located and hence the drift in the bond distances of the parties involved. In this case, the C=O, C-N and C-O distances of Isn and HPip molecules have been analyzed and compared with the values from the anionic Pip ligand present in the complexes, which suggested for both of them a neutral charge (Table 1). In addition, the ΔpKa between both, the conjugated acid of Isn (pK_a_ = 3.61), and HPip (pK_a_ = 4.35) has a value of −0.74 and thus, they are prospective candidates for generate a cocrystal [16].

**Figure 1:**
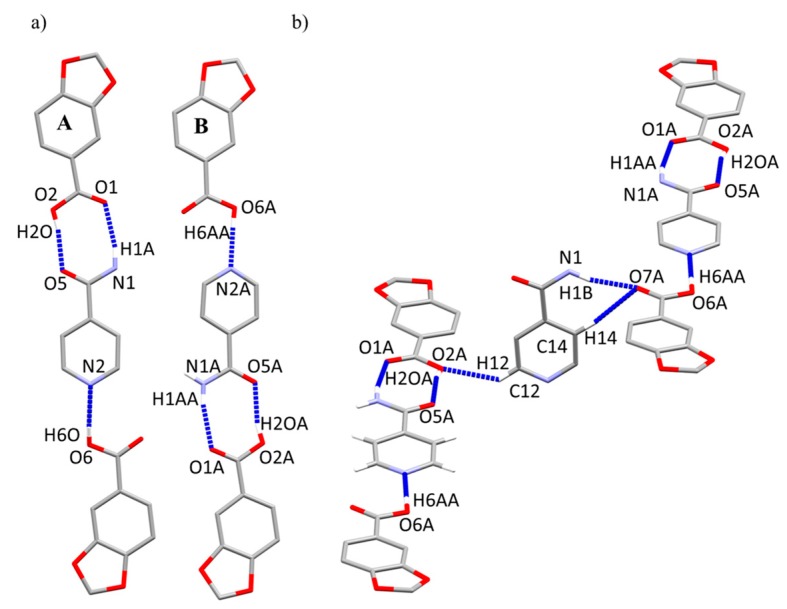
Basic molecules (BSMs) composed by two HPip and one Isn subunits (left, ***A***; right, ***B***) present in compound **2**. (**a**) interactions forming the trimeric BSM (***A***, ***B***). (**b**) Intermolecular interactions between the BSMs.

The basic structural motifs (BSMs) [31], also called long-range synthon Aufbau modules (LSAMs), are two crystallographically independent trimeric large synthons (***A*** and ***B***) constructed by three subunits—two HPip and one Isn. These BSMs are fastened through basic molecules (BSMs) [32]. Those are two small synthons: a type III dimeric square motif (N-H_Isn_···O=C_HPip_; O-H_HPip_···O=C-N-H_Isn_) and a type I dimeric chain motif (O-H···N_Isn_) with distances similar to other reported cocrystals (Table 2) (Figure 1a). In contrast, type II motifs (amide···amide) are not present, probably induced by the MeOH solvent, which is polar enough to break this interaction [33]. Moreover, Isn molecule holds together two adjacent HPip units, from contiguous BSMs, *via* the remaining amidic H_anti_ and the H_ortho_ and H_meta_ from opposite sides. Protons at the same flank merge with the same oxygen atom of the carbonyl, while the H_ortho_ pyridil interacts with the protonated oxygen atom of other HPip molecule (Table 2) (Figure 1b).

**Figure 2:**
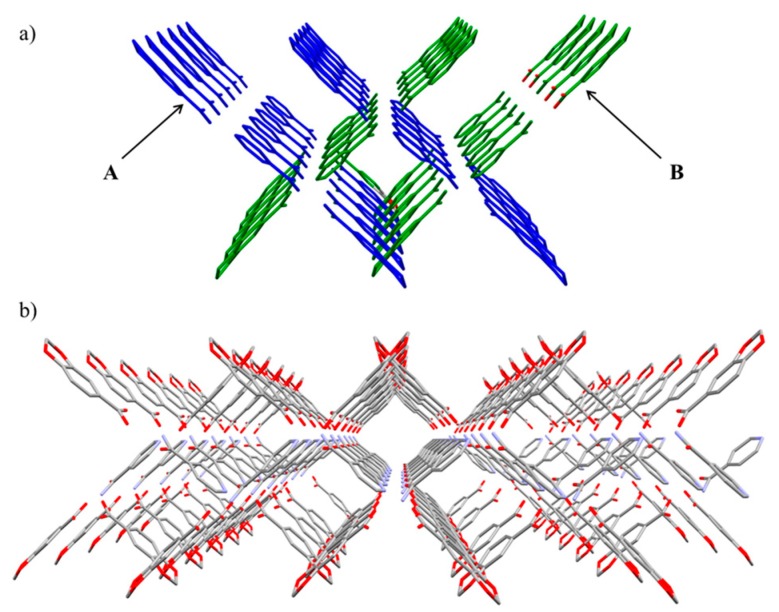
(**a**) α-helix shape through the assembly of ***A*** and ***B*** chains. (**b**) *b* axis view of the 2D layers along the *ac* plane.

BSMs are disposed along the (1 0 1/2) and (1/2 0 3/4) planes (***A*** and ***B***, respectively), and each one of them associates with a closely packed α-helix shape (Figure 2a). These chains are joined *via* the central Isn units, which forms 2D grid shape layers parallel to the *ac* plane (Figure 2b).

### 2.3. Synthesis and Characterization of the Coordination Complexes **3** and **4**

The synthesis of [Cu(Pip)_2_(Isn)_2_] (**3**) and {[Cu_3_(Pip)_2_(OAc)_2_(μ-Isn)_2_(Isn)_2_(μ-OCH_3_)_2_(MeOH)_2_] 2MeOH}_n_ (**4**) was performed mixing **1** and Isn in a 1:7 molar ratio with MeOH as solvent and under reflux conditions for a week. In these conditions, both compounds simultaneously crystallized in a 1.5:1 proportion (violet (**3**) and green (**4**) crystals) and they were mechanically separated. Compound **3** was successfully isolated under reflux conditions and modifying the compound **1**:Isn (1:2) molar ratio. Compound **3** was recrystallized in a MeOH/pentanol (1:1) mixture yielding single crystals of compound [Cu(Pip)_2_(Isn)_2_]·C_5_H_11_OH (**3a**). Unfortunately, **4** could not be obtained as a pure product changing the reaction conditions. Compounds **3** and **4** were characterized by EA, FTIR-ATR, and UV-Vis spectroscopies. The violet crystals of **3** from the mixture were characterized by EA and FTIR-ATR spectroscopies, while single crystals of **3a** were characterized by FTIR-ATR spectroscopy. Complex **4** unavoidably suffers a loss of the occluded MeOH molecules after manipulations required for the EA. In addition, the structure of **3a** and **4** was elucidated by single crystal X-ray diffraction method.

The FTIR-ATR spectra of compounds **3**–**4** (Figures S4–S6) display two pairs of bands at 1557 cm^−1^ (**3**) and 1602 cm^−1^ (**4**) and at 1435 cm^−1^ (**3**) and 1387 cm^−1^ (**4**) assigned to [ν_as_(COO)] and [ν_s_(COO)], respectively, from the carboxylate groups. The difference between these bands (Δ = ν_as_(COO) − ν_s_(COO)) [34] is 121 (**3**) and 215 (**4**), attributable to a chelate and a monodentate coordination mode, respectively. The ν(C=O)_Isn_ band is present at 1694 cm^−1^ in compound **3**, while in **4**, it is split in two at 1696 and 1631 cm^−1^, which suggests the presence of two different Isn ligands. Likewise, the [δ(O=CN)] vibrational mode is identified at 629 cm^−1^ (**3**) and at 627 and 582 cm^−1^(**4**). Additional bands as ν(N-H), δ_ip_(C-H) and δ_oop_(C-H) are also assigned (see exp. section) [28]. If the FTIR-ATR of **3** is compared to **3a**, the unique difference arises in the solvent region in which the alcohol group of the pentanol molecule promoted the formation of broader bands overlapped with those deriving from the amino group (Figure S5).

The electronic spectra of compounds **3** and **4** have been recorded in MeOH as solvent. All spectra show one band in the visible region, characteristic of d^9^ Cu(II) complexes which is assigned to the ^2^E_g_ → ^2^T_2g_ electronic transition (Figure S7) [35]. Compound **3** has its λ_max_ at 713 nm (ε = 77 M^−1^ × cm^−1^), while **4** displayed this d–d electronic transition single band at 706 nm (ε = 404 M^−1^ × cm^−1^). The high ε value of **4** respect to **3** could be explained by the larger number of Cu(II) centers in the polymeric array.

### 2.4. Crystal and Extended Structure of Compound ***3a***

**Figure 3:**
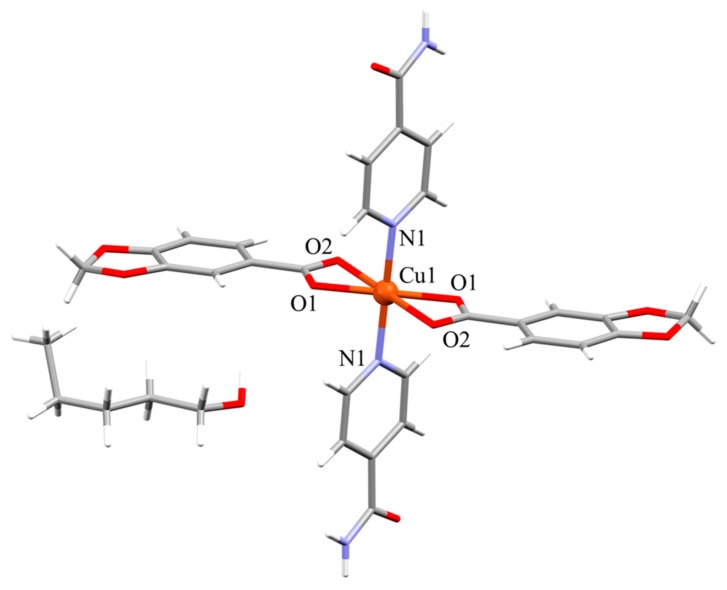
Molecular structure of compound **3a**.

Compound **3a** crystallizes in the triclinic P$\bar{1}$ space group. It has a monomeric structure with a [CuO_4_N_2_] *core* that is composed by two pairs of Pip and Isn ligands (Figure 3). The average twist angle (*ata*) is a useful tool to differentiate between a trigonal prismatic and an octahedral geometry but it must be noted that *ata* value does not give information about how much the geometry is deviated. If this value is closer to 60° the geometry will be octahedral while for values proximate to 0° the geometry probe to be trigonal prismatic [36,37].

The *ata* value for this complex is 60° being the three torsion angles: O2-Cg1-Cg2-O1, −79.26°; N1-Cg1-Cg2-O2, −46.55° and O1-Cg1-Cg2-N1, −54.19°. This six coordinated Cu(II) ion has a distorted octahedral geometry with two chelate Pip units and two monodentate Isn ligands. The axial positions of the octahedron are occupied by the oxygen chelate atoms with the longer Cu-O distance at 2.5069(16) Å, which is usually exhibited by d^9^ Cu(II) ions due to the Jahn-Teller effect [38,39], while the remaining oxygen atoms of the chelate Pip units and the nitrogen atoms of the Isn ligands sets the equatorial plane (Table 3). The chelate coordination mode of the Pip ligands promotes the distortion of the octahedral geometry resulting in angles ranging from 57.69(6)° to 122.31(6)°, and the metal node is out of the equatorial plane (0.522 Å) toward the apical site.

**Figure 4:**
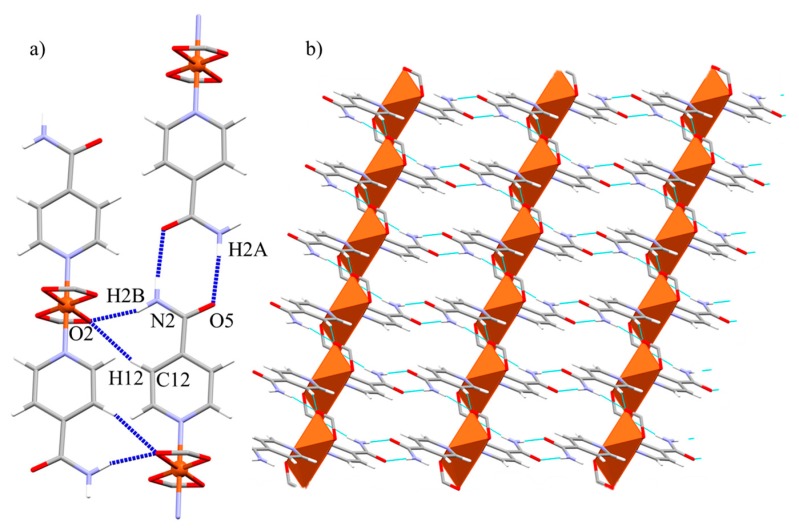
(**a**) Representation of the intermolecular interactions promoted by **3a**. Only carboxylate group of Pip units are shown for clarity. (**b**) View of the 2D nets generated by the Isn···Isn and Isn···Pip interactions along the *ab* plane.

The monomeric units of **3a** are held together *via* two double hydrogen bond interactions (Table 4). The main pair of interactions is the Isn···Isn pattern; the amide moieties engage in a head-to-head disposition, with carbonyl and amide groups pointing in opposite directions. This cooperation expands the structure through the [110] direction forming 1D chains. The other set of interactions are promoted by the furthest coordinated carboxylate oxygen atom of the Pip units that interacts with both hydrogen atoms from the same side of the Isn ligand throughout the [100] direction: one belongs to the amine moiety (N2-H2B···O2), and the other derives from the aromatic C-H_meta_ (C12-H12···O2) (Figure 4a). These two groups of interactions form 2D layers along the *ab* plane (Figure 4b). 

### 2.5. Crystal and Extended Structure of Compound ***4***

**Figure 5:**
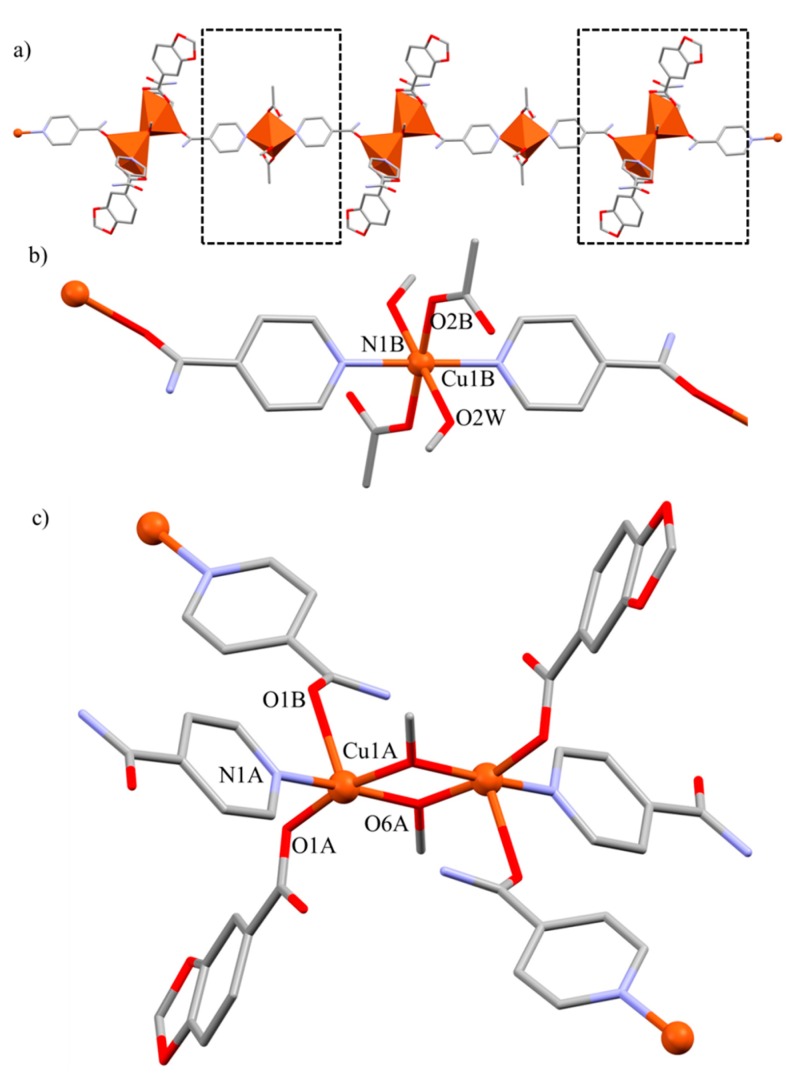
Molecular structure of compound **4**. (**a**) Polymeric 1D chain along the [$\bar{1}$ 11] direction. In detail, a view of (**b**) monomeric units and (**c**) dimeric units of the polymeric array. Hydrogen atoms are omitted for clarity.

Compound **4** crystallizes in the triclinic P$\bar{1}$ space group. It presents a 1D polymeric structure composed of alternated monomeric and dimeric units, which expands through the [$\bar{1}$ 11] direction (Figure 5a). In this polymer, there are three Cu(II) centers with different arrays and coordination environments, displaying both [CuO_4_N_2_] and [CuO_4_N] *cores*. The first one pertains to monomeric units, in which the *O* atoms belong from two acetate and two MeOH molecules while the *N* atoms derive from two Isn ligands (Figure 5b). In the second *core*, the *O* atoms stem from one monodentate Pip unit, two methoxy and one carbonyl group from an Isn unit. The remaining *N* atoms pertain to two different Isn ligands. These methoxy groups frame the dimeric unit *via* a μ_2_-η^1^-*O* coordination mode (Figure 5c). The linkage of the monomeric and dimeric units for the polymeric growth, results from the bidentate bridging μ_2_-η^1^-η^1^-*N*, *O* coordination mode of the Isn ligand. It simultaneously coordinates to the monomeric Cu(II) ion by the pyridyl *N* atom and held together *via* coordination of the carbonyl O_Isn_ atom to one Cu(II) center of the dimeric unit.

All the lengths and angles (Table 5) are in range of other carboxylate and pyridil-containing Cu(II) compounds [20]. These dimeric units display a distorted square pyramidal geometry with a τ value of 0.03 [40]. The hindered disposition of the carbonyl O_Isn_ atom in the dimeric arrays forces the geometry at the apical position. At the same time, the μ_2_-η^1^-*O* coordination modes of the methoxy groups drift the equatorial angles and promote its deviation from the perfect square pyramid (the apical-equatorial angles range from 84.80(10)° to 104.73(10)°). The Isn molecule has a more basic pK_b_ value (10.87) respect to other common pyridine derivatives (2-benzylpyridine, 5.13; 3-phenylpyridine, 4.8; 4-acetylpyridine, 3.51), which can promote the formation of methoxide molecules that drive the arrangement of the polymeric array. The additional reflux period in combination with the basic pK_b_ of the Isn ligand could drive the MeOH groups deprotonation, and their consequent coordination to the Cu(II) ions in **4**.

A further consideration is a large Cu···O bond length of 2.672 Å, which would provide a pseudo-octahedral geometry to the Cu(II) center of the dimeric unit. This distance between the metal ion and the uncoordinated carboxylate oxygen atom of the Pip unit seems to be too long to be considered as a Cu-O bond but not to the extent of being negligible. Other reported longer distances have been previously taken into consideration, as in the case of catena-(bis(μ-D-Hydrogen malato)-copper(II) dihydrate [41]. Different from most of complexes containing Cu(II) and Isn found in the literature, to the best of our knowledge, there is only one reference in which Isn synthon avoids the supramolecular amide···amide pattern at expense of the coordination of the amidic oxygen atom [42].

**Figure 6:**
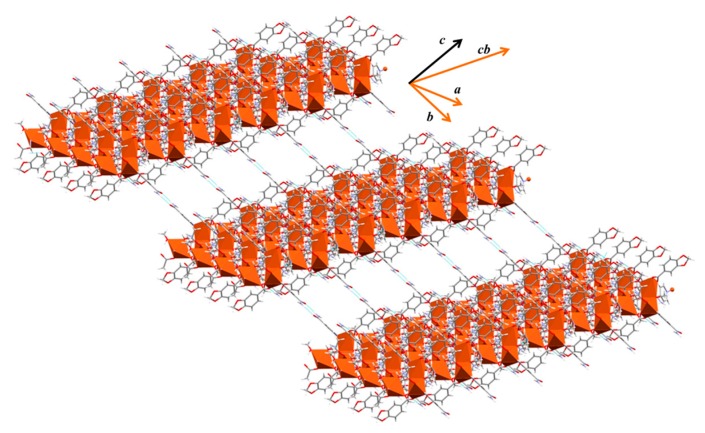
Supramolecular 3D expansion of compound **4**.

The supramolecular structure of **4** is based on the Isn ligand, which acts as a pillar of the expansion joining 1D chains and forming the 3D net (Figure 6). These polymeric chains are non-aligned but consecutively disposed in an alternate monomeric-dimeric manner.

**Figure 7:**
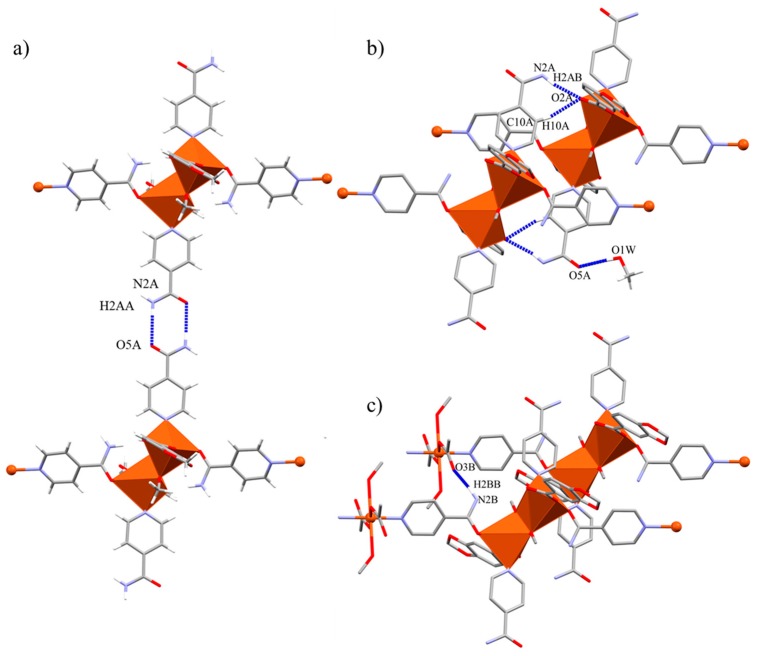
Hydrogen bond interactions present in compound **4**. (**a**) Amide-amide association along the *b* axis. (**b**) N-H_anti_/C-H_ortho_···O=C(Pip) interaction between Isn and Pip ligands through the *bc* plane. (**c**) N-H_anti_ ···O=C(OAc) interaction promoted by bridging Isn ligand.

The monodentate Isn ligand of the dimeric arrays associate the chains by two hydrogen bond interactions. The mentioned amide···amide pattern holds together dimeric arrays through the *b* direction (Figure 7a), and this Isn units also participate in the expansion *via* a double N-H_anti_/C-H_ortho_···O=C interaction with a monodentate Pip unit of other dimeric array in the *bc* direction. Besides, the carbonyl of this Isn ligand also forms an O···H-C interaction with the occluded MeOH molecule (Figure 7b). The final 3D net is supported by the bidentate bridged Isn linkers, which interact along the *a* axis by means of their N-H_anti_ with the non-coordinated carbonyl oxygen atom of the monodentate OAc units pertaining to the monomeric arrays (Table 6) (Figure 7c).

### 2.6. Hirshfeld Surfaces Analysis and Energy Frameworks Calculations

Hirshfeld surfaces analysis of all the structures, energy frameworks and lattice energy calculations of complexes **2** and **3a** have been performed with CrystalExplorer 17.5 [43]. All the surfaces have been calculated at an isovalue of 0.5 e × au^−3^. The crystal structures of the (HPip)_2_(Isn) (**2**) adduct and **3a** have been examined by energy framework based on TONTO, using CE-B3LYP(d,p) molecular wave-functions, calculated using .cif files obtained from the single crystal X-ray diffraction data. The disorder present in **3a** has been identified, and the fractional occupancy has been corrected to 1 in the .cif file. It has not been possible to perform the same calculations with **4** owing to its polymeric structure.

Hirshfeld surface, in combination with 2D fingerprint plot, are powerful graphical tools to evaluate intra- and intermolecular interactions present in crystal structures. The surface mapping facilitates their identification while the fingerprint plot outlines the distances between the atoms involved in these interactions. All these calculations required the generation of the Hirshfeld surface with electron density mapping to construct the wave-function of each selected molecule. Owing to **2** being a multi-component crystal, this surface mapping has been performed for each unique molecule in the unit cell (four HPip and two Isn), verifying that there is one computed wave-function for each unique molecule. Hence, energies were calculated separately for each unique molecule in the unit cell (those from the asymmetric unit), and the energy framework was constructed based on crystal symmetry.

In the same way, Hirshfeld surface with electron density mapping and energy calculation of **3a** have been performed for each unique molecule in the unit cell (one monomer and one pentanol molecule), and the energy frameworks were constructed based on crystal symmetry. The selection of the appropriate radius for the energy calculation depends on the polarity of the parts involved in the interactions. More polar groups will need a bigger radius, while less polar molecules require a smaller one. Pairwise interaction energies between molecules were computed considering radius of 20.0 Å for **2** and **3a** from the centroid of the selected molecule to an atom belonging to its nearest neighbor. Energy framework is a unique tool to visualize the supramolecular architecture of crystal structures. The total energy (E_tot_) is divided into electrostatic (E_ele_), polarization (E_pol_) and dispersion (E_dis_) components in which cylinders represent the relative strength of the molecular packing based on a scale factor. The comparison between these components is only possible if the scale factor is the same. For **2** and **3a**, the scale factor has been fixed at 240.

The main interactions present in compound **2** are identified and highlighted by Hirshfeld surface analysis (Figure S8a). The shape of the 2D fingerprint plot as well as the percentage of surface implied in the interactions suggests a difference between those which held the ***A*** and ***B*** BSMs (Figure S8b). This data was not sufficiently clear, so a further study including energy calculations was required (*vide infra*). By comparison, the main interactions and the percentage of surface implied present in compounds **3a** and **4** are pinpointed in the Figure S9. In both, the majority involved belongs to the Isn ligand (amide···amide and N-H_anti_/C-H···O interactions) and therefore have similar values of surface area implied in them.

**Figure 8:**
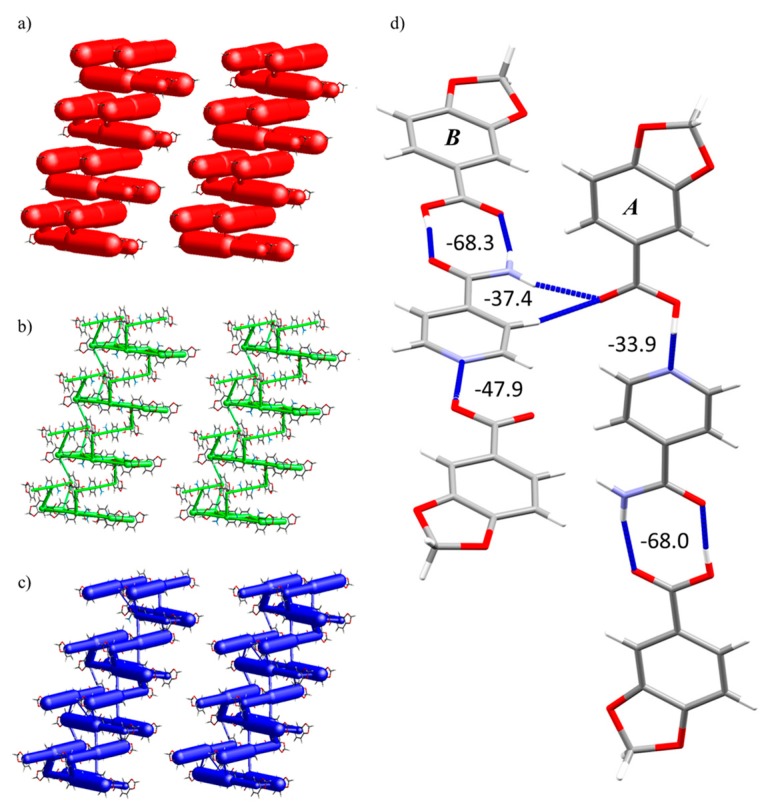
Energy framework diagram (E_ele_, E_dis_ and E_tot_) for compound **2**. All diagrams use the same energy cylinder scale of 240, and the energy threshold is 6.00 kJ·mol^−1^. (**a**) Electrostatic energy. (**b**) Dispersion energy. (**c**) Total energy. (**d**) BSMs ***A*** and ***B*** pinpointing the main interactions and their energy values in kJ·mol^−1^.

Energy frameworks of **2** exhibited that the most important energy component is electrostatic (Figure 8a). These attractive forces derive from the amide···acid heterosynthon, while the dispersion energy is comparatively low and is mainly generated by the interaction between HPip units itself (Figure 8b). The total energy framework shows the association of BSMs in pairs that are stacked along the b axis (Figure 8c). The assembly of this cocrystal is dominated by O-H···N and N-H···O hydrogen bonds from the heterosynthons. The strongest associations in both ***A*** and ***B*** BSMs arise from the amide···acid dimeric square motifs (−68.3 kJ·mol^−1^ for ***B*** and −68.0 kJ·mol^−1^ for ***A***) with similar but not identical energy values. Followed by the N···H-O chain motifs with energy values of −47.9 kJ·mol^−1^ (***B***) and −33.9 kJ·mol^−1^ (***A***) (Figure 8d). 

As suggested by the 2D fingerprint plot with percentage values, there was a difference in the interaction that associates the BSMs. The energy of the N···H-O interaction in ***A*** and ***B*** presented a difference of ΔE_***B***-***A***_(N···H-O) = 14.0 kJ·mol^−1^, which is not negligible and is probably driven by the neighboring interaction in which the carbonyl group participates. These two BSMs are fastened by the central Isn unit, which forms this mentioned C-H/N-H···O double interaction with an acid of the neighboring BSMs with an energy value of −37.4 kJ·mol^−1^.

The computed lattice energy (*E_lat_*) value of each unique molecule has been calculated according to Equation (1), in which *N_i_* is the number of molecule pairs in the cluster with that particular interaction energy and *E_i_* is the interaction energy value.

(1) $$E_{lat}=\frac{1}{2}\overset{n}{\underset{i=1}{\sum }}\left(N_{i}\cdot E_{i}\right)$$

The average *E_lat_* value of each HPip and Isn coformers has been calculated, and the crystal *E_lat_* is computed considering the 2:1 (HPip:Isn) molar ratio. The crystal structure *E_lat_* value of **2** is −362.8 kJ·mol^−1^, which is comparable to other reported energies of multicomponent cocrystals [44].

The energy frameworks of **3a** are also driven by the electrostatic component, in which weaker destabilizing energies (1.0 and 2.2 kJ·mol^−1^) belong to the occluded pentanol molecules (Figure S10a), while the most remarkable dispersion energy is promoted by the parallel Pip ligands along the *c* axis (Figure S10b). When compared to **2**, the dispersion energy of **3a** is more significant and plays a relevant role in this system. This is induced by the restriction on the orientation of the Pip ligands caused by the coordination to the metal center.

The total energy frameworks confirm that the main interactions are those from Isn as mentioned in the structural description (Figure S10c). The amide···amide pattern stacks the monomeric units along the [110] with an energy value of −63.5 kJ·mol^−1^ and these chains are assembled through the N-H/C-H···O interactions with two energy values of −23.3 and −21.6 kJ·mol^−1^. The crystal structure *E_lat_* value is −414.6 kJ·mol^−1^ and it has also been calculated according to Equation (1).

## 3. Conclusions

We successfully isolated three different isonicotinamide based compounds by modifying the reaction conditions. The crystal structure elucidation of **2**, **3a**, and **4** allowed ascertaining the formation of a cocrystal (**2**), a monomer (**3a**), and an unusual coordination polymer (**4**). Isn ligand can acts as either hydrogen bond donor or acceptor or both by strong N···H-O and N-H···O interactions. Likewise, it has a potential role as mono- or bidentate linker coordinating to the Cu(II) center *via* *N*- or *N,O*-atoms, and these tendencies could promote the formation of the mixtures.

Compound **2** is constructed by type I and type III synthons instead of type II, which is the most common association in Isn-containing systems. This could be driven by the strong hydrogen bond donor solvent (MeOH) used in the crystallization process which probably dissociated the weaker type II homomeric synthon present in Isn and facilitated the formation of the stronger type III interaction. The union of both, Isn and HPip, fulfill the pK_a_ rule and supports the formation of a cocrystal instead of a salt.

In the two complexes **3a** and **4**, both Pip and Isn ligands participated in the growth of the final scaffolds, but Isn outstands as the keystone in the formation of the products. In the precursor structure (**1**), the Pip units act as bidentate bridged while in **3a** and **4**, they exhibited a chelate and a monodentate coordination mode, respectively. Likewise, the Isn unit also adopts two coordination modes (monodentate (**3a**) or bidentate bridged (**4**)). The additional reflux period, in combination with the basic pK_b_ of the Isn ligand, could drive the MeOH groups deprotonation and their consequent coordination to the Cu(II) ions in **4**. It is noteworthy that Isn ligand has been the driving unit of both monomeric and coordination polymer, designing different molecular and supramolecular networks and downgrading Pip to an auxiliary ligand.

Hirshfeld surface analyses pinpointed the role of Isn as synthon. Furthermore, energy framework calculations distinguished Isn as the driving cohesive force, while HPip itself mainly participates in the London dispersion forces. Lattice energy calculation showed a notable value as a consequence of the large number of interactions promoted by the Isn ligand.

The formation of the cocrystal has enabled us to easily recognize the role of Isn as synthon and facilitated the better understanding of the driving forces in those systems.

## 4. Experimental Section

Cu(II) acetate monohydrate (Cu(OAc)_2_·H_2_O), 1,3-benzodioxole-5-carboxylic acid (piperonylic acid, HPip), and isonicotinamide (Isn); methanol (MeOH) and pentanol (C_5_H_11_OH) for recrystallization were used as solvents and were purchased from Sigma-Aldrich (Darmstadt, Germany). Deuterated dimethylsulfoxide (dmso-*d*_6_) was used for the NMR experiments and was purchased from Eurisotop (Saint-Aubin, France). All of them were used without further purification. The [Cu(μ-OAc)(μ-Pip)(MeOH)]_2_ (**1**) was previously synthesized in our research group [26,27].

Melting point was measured on an MPM-H2 KLEINFELD (Gehrden, Germany) melting point meter apparatus. Elemental analysis (C, H, N) were carried out on a Thermo Scientific (Waltham, MA, USA) Flash 2000 CHNS Analyzer. The FTIR-ATR spectra were recorded on a Perkin Elmer (Waltham, MA, USA) spectrometer, equipped with a universal attenuated total reflectance (ATR) accessory with diamond window in the range 4000–550 cm^−1^. ^1^H-NMR and ^13^C{^1^H}-NMR spectra were recorded on an NMR-FT Bruker400 (Karlsruhe, Germany) MHz spectrometer in dmso-*d*_6_ solution at room temperature (r.t.). All chemical shifts (δ) are given in ppm relative to TMS as internal standard. The electronic spectra in solution of MeOH (≈1.0 × 10^−3^ M) were run on an Agilent HP 8453 UV-Vis spectrophotometer with a quartz cell having a path length of 1 cm in the range of 500–900 nm. Spectroscopic characterization details of all compounds are in the Supplementary Materials (Figures S1–S7).

Synthesis of (HPip)_2_(Isn) (**2**) and [Cu(Pip)_2_(Isn)_2_] (**3**): A methanolic solution (25 mL) of **1** (98.9 mg, 0.155 mmol) was added dropwise to a methanolic solution (15 mL) of Isn (38.1 mg, 0.312 mmol) and was stirred for 2 h at r.t. A mixture of colorless and violet crystals was obtained by slow evaporation of the solution on air for 10 days.

Synthesis of (HPip)_2_(Isn) (**2**): A methanolic solution (15 mL) of HPip (100 mg, 0.602 mmol) was added dropwise to a methanolic solution (10 mL) of Isn (36.7 mg, 0.300 mmol) and was stirred for 4h at r.t. After that, reaction was concentrated until half of the volume. Suitable colorless crystals were obtained by slow evaporation of the solution on air for three days. Single crystals were filtered and washed with cold diethyl ether.

**2.** Yield: 49.5 mg (57%). Mp 193–194 °C. Elem. Anal. Calc. for C_22_H_18_N_2_O_9_ (454.39 g/mol): C 58.15; H 3.99; N 6.17. Found: C 58.02; H 3.84; N 6.14. FTIR-ATR (cm^−1^): 3321(m) [ν(N-H)], 3140(m) [ν(O-H)], 3075(w), 3072(w) [ν_ar_(C-H)], 2907(w) [ν_al_(C-H)], 2791(w), 2650-2283(w), 1680(s) [ν(C=O)], 1620(m), 1551(m) [ν(C=C/C=N)], 1500(m), 1482(w), 1431(w), 1409(s), 1361(m) [δ(C=C/C=N)], 1280(w), 1254(w), 1233(m), 1164(m) [ν(C-O-C)], 1108(m), 1032(m), 1023(m) [δ_ip_(C-H)], 906(m), 763(m) [δ_oop_(C-H)], 707(w), 674(w), 643(s) [δ(O=CN)], 583(w). ^1^H-NMR (360 MHz, DMSO-*d*_6_, Me_4_Si, 298 K): δ = 8.71 [2H, d, ^3^*J* = 5.8 Hz, CH-C***H***-N]_Isn_, 8.24 [1H, br, OCN***H***H-C]_Isn_, 7.76 [2H, dd, ^3^*J* = 4.5 Hz; ^4^*J* = 1.5 Hz, C***H***-CH-N]_Isn_, 7.73 [1H, br, OCNH***H***-C]_Isn_, 7.54 [2H, dd, ^3^*J* = 8.1 Hz; ^4^*J* = 1.5 Hz, C***H***-CH-C-O]_HPip_, 7.35 [2H, d, ^4^*J* = 1.5 Hz, C***H***-C-O]_HPip_, 6.99 [2H, d, ^3^*J* = 7.9 Hz, CH-C***H***-C-O]_HPip_, 6.11 [4H, s, O-C***H***_**2**_-O]_HPip_. ^13^C{^1^H}-NMR (360 MHz, DMSO-*d*_6_, Me_4_Si, 298 K): δ = 166.89 [O_2_***C***-C]_HPip_, 166.62 [O***C***NH_2_-C]_Isn_, 151.29 [CH-CH-***C***-O]_HPip_, 150.34 [CH-***C***H-N]_Isn_, 147.63 [CH-***C***-O]_HPip_, 141.46 [O***C***NH_2_-***C***]_Isn_, 125.15 [***C***H-CH-C-O]_HPip_, 124.82 [O_2_C-***C***]_HPip_, 121.65 [***C***H-CH-N]_Isn_, 108.99 [***C***H-C-O]_HPip_, 108.19 [CH-***C***H-C-O]_HPip_, 102.10 [O-***C***H_2_-O]_HPip_.

Synthesis of [Cu(Pip)_2_(Isn)_2_] (**3**): A methanolic solution (25 mL) of **1** (100 mg, 0.156 mmol) was added dropwise to a methanolic solution (15 mL) of Isn (39.0 mg, 0.319 mmol) and was stirred for 4h under reflux conditions. Solution turned dark blue, and a blue powder precipitated. The solid was filtered, washed with 5 mL of cold MeOH, and dried under vacuum. The powder was recrystallized in pentanol, and the resulting solution remained exposed to air until violet crystals of [Cu(Pip)_2_(Isn)_2_]·C_5_H_11_OH (**3a**) were grown by slow evaporation after four days.

**3.** Yield: 87.3 mg (88%). Elem. Anal. Calc. for C_28_H_22_CuN_4_O_10_ (638.05 g/mol): C 52.71; H 3.48; N 8.78. Found: C 52.82; H 3.64; N 8.64. FTIR-ATR (cm^−1^): 3333(br) [ν(N-H)], 3165(br) [ν(N-H)], 3072(w) [ν_ar_(C-H)], 2964-2860(br) [ν_al_(C-H)], 1694(m) [ν(C=O)], 1630(m), 1617(m), 1557(m) [ν_as_(COO)], 1543(m) [ν(C=C/C=N)], 1498(m), 1435(m) [ν_s_(COO)], 1384(s) [δ(C=C/C=N)], 1343(m), 1247(s), 1165(m) [ν(C-O-C)], 1108(m), 1067(m), 1035(s) [δ_ip_(C-H)], 938(m), 924(m), 805(m) [δ_oop_(C-H)], 772(s) [δ_oop_(C-H)], 722(m), 682(m), 651(m), 629(m) [δ(O=CN)], 590(m). UV-Vis (MeOH, 1.10·10^−3^ M) λ_max_ (ε) = 713 (77) nm.

Compound **3a** presented the same FTIR-ATR spectrum as **3** with small differences in the 3400–2900 cm^−1^ region due to the presence of ν(O-H) signals attributable to pentanol molecules.

Synthesis of [Cu(Pip)_2_(Isn)_2_] (**3**) and {[Cu_3_(Pip)_2_(OAc)_2_(μ-Isn)_2_(Isn)_2_(μ-OCH_3_)_2_(MeOH)_2_] ·2MeOH}_n_ (**4**): A methanolic solution (25 mL) of **1** (100 mg, 0.156 mmol) was added dropwise to a methanolic solution (15 mL) of Isn (127 mg, 1.04 mmol) and was stirred vigorously under reflux conditions for a week. Solution was evaporated until half of the volume and kept at 4 °C for a month until simultaneous violet (**3**, Yield: 51.4 mg (26%)) and green (**4**, Yield: 34.1 mg (52%)) crystals were formed. These crystals were mechanically separated.

**4.** Elem. Anal. Calc. for C_48_H_54_Cu_3_N_8_O_20_ (1253.62 g/mol): C 45.99; H 4.34; N 8.94. Found: C 45.67; H 4.29; N 9.18. FTIR-ATR (cm^−1^): 3435(w), 3333(br) [ν(N-H)], 3276(w), 3211 (br) [ν(N-H)], 3071(w) [ν_ar_(C-H)], 2992-2830(w) [ν_al_(C-H)], 1696(m) [ν(C=O)], 1631(m), 1602(s) [ν(C=O)], 1554(m) [ν_as_(COO)], 1504(m) [ν(C=C/C=N)], 1490(m), 1436(s), 1418(s) [δ(C=C/C=N)], 1387(s) [ν_s_(COO)], 1258(s), 1243(m), 1221(m), 1170(m) [ν(C-O-C)], 1113(m), 1079(m), 1067(m), 1033(s) [δ_ip_(C-H)], 1015(m) [δ_ip_(C-H)], 935(m), 920(m), 889(m), 856(m), 805(m), 774(s) [δ_oop_(C-H)], 765(m) [δ_oop_(C-H)], 732(m), 683(s), 627(s) [δ(O=CN)], 582(s) [δ(O=CN)], 565(m). UV-Vis (MeOH, 9.90·10^−4^ M) λ_max_ (ε) = 706 (404) nm.

## 5. Crystallographic Data

A colorless (**2**), a violet (**3a**), and a green (**4**) prism-like specimens were used for the X-ray crystallographic analysis. The X-ray intensity data were measured on a D8 Venture system equipped with a multilayer monochromator and a Mo microfocus (λ = 0.71073 Å). For **2**–**4**, the frames were integrated with the Bruker SAINT software package using a narrow-frame algorithm. All hydrogen atoms were refined using a riding model (AFIX) with an isotropic temperature factor equal to 1.2, the equivalent temperature factor of the atom to which are linked and thus, the bond lengths of X–H were fixed. The structures were solved and refined using the Bruker SHELXTL Software package and refined using SHELX (version 2018/3) [45]. For **2**–**4**, the final cell constants and volumes are based upon the refinement of the XYZ-centroids of reflections above 20 σ(I). Data were corrected for absorption effects using the Multi-Scan method (SADABS). Crystal data and relevant details of structure refinement for compounds **2**–**4** are reported in Table 7. CCDC 1944986 (**2**), 1944988 (**3a**), and 1944987 (**4**) contain the Supplementary Data of this paper. Molecular graphics were generated using Mercury 4.1.2 software [46,47] with POV-Ray package [48]. Color codes for all molecular graphics: orange (Cu), red (O), blue (N), dark grey (C), and white (H).

## Figures and Tables

**Table 1:** Comparative of bond lengths (C=O, C-N, C-N) between cocrystal and monomer and polymer compounds.

	2		3a		4	
	BSM *A*	BSM *B*				
Bond	Length (Å)	Distance (Å)	Bond	Length (Å)	Bond	Distance (Å)
**C=O**_Isn_	1.253(4)	1.241(5)	**C=O**_Isn_	1.236(2)	**C=O**_Isn coord_	1.234(4)
**C=O**_HPip_···N_py_	1.233(4)	1.226(4)	**C=O**_Pip_	1.281(3)	**C=O**_Isn_	1.240(5)
**C=O**_HPip_···H-N_amide_	1.217(5)	1.228(5)	**C=O**_Pip_	1.250(3)	**C=O**_Pip_	1.330(5)
**C-N**	1.321(4)	1.326(4)	**C-N**	1.328(3)	**C=O**_Pip_	1.317(6)
**C-OH**_HPip_···N_py_	1.300(5)	1.314(4)			**C-N**_Isn coord_	1.276(6)
**C-OH**_HPip_···H-N_amide_	1.324(4)	1.318(4)			**C-N**	1.257(5)

Selected distances are highlighted in bold.

**Table 2:** Supramolecular interactions present in compound **2**.

Intra-BSM *A*				
	H···A (Å)	D···A (Å)	D-H (Å)	>D-H···A (°)
O6-H6O···N2	1.916	2.611	0.840	139.37
N1-H1A···O1	1.979	2.840	0.881	165.44
O2-H2O···O5	1.835	2.645	0.840	161.52
Intra-BSM ***B***				
O6A-H6AA···N2A	1.778	2.615	0.841	173.73
N1A-H1AA···O1A	1.969	2.831	0.881	166.02
O2A-H2OA···O5A	1.819	2.634	0.840	163.11
Inter-BSM ***A***				
N1-H1B···O7A	2.033	2.880	0.880	161.48
C14-H14···O7A	2.342	3.279	0.950	168.81
C12-H12···O2A	2.374	3.099	0.950	132.81
Inter-BSM ***B***				
N1A-H1AB···O7	2.031	2.879	0.880	161.40
C13A-H13A···O2	2.376	3.097	0.950	132.40
C11A-H11A···O7	2.344	3.281	0.950	168.84

**Table 3:** Selected bond lengths (Å) and bond angles (°) for compound **3a**.

Bond Length (Å)			
Cu(1)-O(1)#1	1.9673(14)	Cu(1)-N(1)#1	1.9988(16)
Cu(1)-O(2)	2.5069(16)	Cu···Cu	8.023
**Bond Angles (°)**			
O(1)#1-Cu(1)-O(1)	180	O(2)-Cu(1)-O(1)	57.69(6)
O(1)#1-Cu(1)-N(1)	89.88(6)	O(2)-Cu(1)-O(1)#2	122.31(6)
O(1)-Cu(1)-N(1)	90.12(6)	N(1)-Cu(1)-O(2)	91.26(6)
N(1)-Cu(1)-N(1)#1	180	N(1)-Cu(1)-O(2)#1	88.74(6)
O(2)-Cu(1)-O(2)#1	180		

#1: −x, −y + 1, −z + 1; #2: −x, 1 − y, 1 − z.

**Table 4:** Selected intermolecular interactions for compound **3a**.

3b	H···A (Å)	D···A (Å)	D-H (Å)	>D-H···A (°)
N2-H2A···O5	2.013	2.887	0.880	172.27
N2-H2B···O2	1.986	2.842	0.880	164.42
C12-H12···O2	2.330	3.251	0.950	163.28

**Table 5:** Selected bond lengths (Å) and bond angles (°) for compound **4**.

Bond Length (Å)			
Cu(1A)-O(6A)	1.936(3)	Cu(1B)-N(1B)#1	2.006(3)
Cu(1A)-O(1A)	1.957(3)	Cu(1B)-O(2W)	2.546(3)
Cu(1A)-O(6A)#2	1.957(2)	Cu(1A)···Cu(1A)	3.052(9)
Cu(1A)-O(1B)	2.395(3)	Cu(1A)···Cu(1B)	10.820(9)
Cu(1A)-N(1A)	2.011(3)	Cu(1A)···Cu(1B)	9.139(9)
Cu(1B)-O(2B)#1	1.964(3)		
**Bond Angles (°)**			
O(6A)-Cu(1A)-O(1A)	167.18(11)	O(1A)-Cu(1A)-O(1B)	84.80(10)
O(6A)-Cu(1A)-O(6A)#2	76.74(11)	O(6A)#2-Cu(1A)-O(1B)	97.61(10)
O(1A)-Cu(1A)-O(6A)#2	93.70(11)	N(1A)-Cu(1A)-O(1B)	90.83(11)
O(6A)-Cu(1A)-N(1A)	94.18(12)	O(2B)#1-Cu(1B)-O(2B)	180.00(15)
O(1A)-Cu(1A)-N(1A)	94.24(12)	O(2B)#1-Cu(1B)-N(1B)	91.01(12)
O(6A)#2-Cu(1A)-N(1A)	168.90(12)	O(2B)#1-Cu(1B)-N(1B)#1	88.99(12)
O(6A)-Cu(1A)-O(1B)	104.73(10)	N(1B)-Cu(1B)-N(1B)#1	180.0
O(1B)-Cu(1A)-O(6A)	104.71(10)	O(1B)-Cu(1A)-N(1A)	90.82(11)

#1: −x + 1, −y, −z + 1; #2: −x, −y + 1, −z + 2.

**Table 6:** Selected intermolecular interactions for compound **4**.

	H···A (Å)	D···A (Å)	D-H (Å)	>D-H···A (°)
N(2B)-H(2BA)···O(6A)	2.14	2.883(4)	0.88	142
O(1W)-H(1W)···O(5A)	1.90	2.739(6)	0.84	173
N(2B)-H(2BB)···O(3B)	2.02	2.791(4)	0.88	145
O(2W)-H(2W)···O(3B)	1.86(7)	2.654(5)	0.81(7)	167(7)
N(2A)-H(2AA)···O(5A)	2.04	2.916(5)	0.88	178
N(2A)-H(2AB)···O(2A)	1.92	2.781(5)	0.88	164

**Table 7:** Crystal and structure refinement data for compounds **2**–**4**.

	2	3a	4
Empirical formula	C_22_H_18_N_2_O_9_	C_33_H_34_CuN_4_O_11_	C_50_H_62_Cu_3_N_8_O_22_
Formula weigh	454.38	726.18	1317.69
*T* (K)	100(2) K	100(2)	100(2)
Wavelength (Å)	0.71073 Å	0.71073	0.71073
System, space group	Triclinic, P $\bar{1}$	Triclinic, P $\bar{1}$	Triclinic, P $\bar{1}$
Unit cell dimensions			
*a* (Å)	9.6976(3)	8.0226(8)	7.8402(3)
*b* (Å)	12.6877(6)	11.4099(12)	13.7091(6)
*c* (Å)	16.1988(7)	11.9187(13)	14.3107(6)
*α* (°)	82.329(2)	1.114(4)	109.2060(10)
*β* (°)	81.3340(10)	96.344(4)	97.8040(10)
*γ* (°)	89.9900(10)	107.632(3)	99.7130(10)
V (Å^3^)	1952.31(14)	988.10(18)	1401.15(10)
*Z*	4	1	1
*D*calc (g × cm^3^)	1.546	1.220	1.562
*µ* (mm^−1^)	0.122	0.609	1.215
*F*(000)	944	377	681
Crystal size (mm^3^)	0.242 × 0.082 × 0.081	0.164 × 0.093 × 0.072	0.251 × 0.071 × 0.046
*hkl* ranges	−11 ≤ h ≤ 12 −16 ≤ k ≤ 16 −21 ≤ l ≤ 21	−11 ≤ h ≤ 11 −16 ≤ k ≤ 16 −17 ≤ l ≤ 17	−9 ≤ h ≤ 8 −15 ≤ k ≤ 15 −16 ≤ l ≤ 16
*2θ* range (°)	2.198 to 28.299	1.959 to 30.583	2.603 to 24.403
Reflections collected/ unique/[Rint]	69395/9659 [R(int) = 0.0953]	51118/6046 [R(int) = 0.0428]	32892/4577 [R(int) = 0.0309]
Completeness to θ = 25.242°	99.9	99.6	99.6
Absorption Correction	Semi-empirical	Semi-empirical	Semi-empirical
Max. and min. transmis.	0.7457 and 0.6910	0.7461 and 0.6872	0.7451 and 0.6975
Refinement method	Full-matrix least-squares on |F|^2^	Full matrix least-squares on |F|^2^	Full matrix least-squares on |F|^2^
Data/restrains/parameters	9659/1/597	6046/0/250	4577/3/382
Goodness of fit on |*F|*^2^	1.039	1.112	1.095
Final *R* indices [*I* > 2σ(*I*)]	R_1_ = 0.0888, wR_2_ = 0.2189	R_1_ = 0.0541, wR_2_ = 0.1690	R_1_ = 0.0444, wR_2_ = 0.1110
*R* indices (all data)	R_1_ = 0.1360, wR_2_ = 0.2559	R_1_ = 0.0591, wR_2_ = 0.1746	R_1_ = 0.0519, wR_2_ = 0.1172
Extinction coefficient	n/a	n/a	n/a
Largest. Diff. peak and hole (e Å^−3^)	1.329 and −0.722	2.077 and −0.717	1.472 and −0.700

## Supplementary Materials

The following are available online at www.mdpi.com/xxx/s1, Figure S1. FTIR-ATR spectra of a. compound (HPip)_2_(Isn) (**2**); b. Isn and c. HPip.; Figure S2. ^1^H-NMR spectra of a. compound (HPip)_2_(Isn) (2); b. HPip and c. Isn.; Figure S3. ^13^C{^1^H}-NMR spectrum of a. compound (HPip)_2_(Isn) (**2**); b. HPip and c. Isn., Figure S4. FTIR-ATR spectrum of compound [Cu(Pip)_2_(Isn)_2_] (**3**).; Figure S5. FTIR-ATR spectrum of compound [Cu(Pip)_2_(Isn)_2_]·(C_5_H_11_OH) (**3a**).; Figure S6. FTIR-ATR spectrum of compound {[Cu_3_(Pip)_2_(OAc)_2_(μ-Isn)_2_(Isn)_2_(μ-OCH_3_)_2_(MeOH)_2_]·2MeOH}_n_ (**4**).; Figure S7. Electronic spectra of compounds **3** and **4** between 500 nm and 900 nm in MeOH solution.; Figure S8. Hirshfeld surface representation and fingerprint plot of **2**. In detail view of a. each molecule which forms the BSMs and b. each intra-BSM interaction with the surface area implied in it.; Figure S9. Hirshfeld surface representation and fingerprint plot of a. **3a** and b. **4**.; Figure S10. Energy frameworks diagram of a. E_ele_, b. E_dis_, c. E_tot_ for compound **3a**. All diagrams use the same energy cylinder scale of 240 and the energy threshold was fixed at 3.0 kJ·mol^−1^ for E_ele_ and 30.0 kJ·mol^−1^ for E_dis_ and E_tot_. Color codes of energy frameworks are red (E_ele_); green (E_dis_); blue (E_tot_) and yellow (destabilizing energies). Complete information about the crystal structure and molecular geometry is available in .cif format as Supporting Information.

## References

[1] Swiegers G.F., Malefetse T.J. (2000). New Self-Assembled Structural Motifs in Coordination Chemistry. Chem. Rev..

[2] Desiraju G.R. (2000). Hydrogen bonds and other intermolecular interactions in organometallic crystals. J. Chem. Soc. Dalton Trans..

[3] (2016). Cambridge Structural Database (CSD) (Version 5.37). https://libguides.caltech.edu/crystallography/CSD#Applications.

[4] Ye B.-H., Tong M.-L., Chen X.-M. (2005). Metal-organic molecular architectures with 2,2′-bipyridyl-like and carboxylate ligands. Coord. Chem. Rev..

[5] Etter M.C. (1990). Encoding and decoding hydrogen-bond patterns of organic compounds. Acc. Chem. Res..

[6] Desiraju G.R. (1995). Supramolecular Synthons in Crystal Engineering—A New Organic Synthesis. Angew. Chem., Int. Ed..

[7] Nangia A., Desiraju G.R., Weber E., Aoyama Y., Caira M.R., Desiraju G.R., Glusker J.P., Hamilton A.D., Meléndez R.E., Nangia A. (1998). Supramolecular Synthons and Pattern Recognition. Design of Organic Solids.

[8] Wang Y.-Y., Shi Q., Shi Q.-Z., Gao Y.-C., Zhou Z.-Y. (1999). Syntheses, characterization and crystal structure of copper(II) α,β-unsaturated carboxylate complexes with imidazole. Polyhedron.

[9] Choudhury A.R., Desiraju G.R., Dikundwar A.G., Dubey R., Duggirala N., Ghogale P.P., Ghosh S., Goswami P.K., Goud N.R., Jetti R.R.K.R. (2012). Polymorphs, Salts, and Cocrystals: What’s in a Name?. Cryst. Growth Des..

[10] Germán-Acacio J.M., Hernández-Ortega S., Aakeröy C.B., Valdés-Martínez J. (2009). Using Lewis acidity differences in chelating ligands to control molecular structure and supramolecular assembly of Cu(II) complexes. Inorg. Chim. Acta.

[11] Ɖakovic M., Jagličić Z., Kozlevčar B., Popović Z. (2010). Association of copper(II) isonicotinamide moieties via different anionic bridging ligands: Two paths of ferromagnetic interaction in the azide coordination compound. Polyhedron.

[12] Ataç A., Yurdakul Ş., Ide S. (2006). Synthesis and vibrational spectroscopic studies of isonicotinamide metal(II) halide complexes. J. Mol. Struct..

[13] Mukherjee A. (2015). Building upon Supramolecular Synthons: Some Aspects of Crystal Engineering. Cryst. Growth Des..

[14] Yenikaya C., Poyraz M., Sari M., Demirci F., İlkimen H., Büyükgüngör O. (2009). Synthesis, characterization and biological evaluation of a novel Cu(II) complex with the mixed ligands 2,6-pyridinedicarboxylic acid and 2-aminopyridine. Polyhedron.

[15] Cruz-Cabeza A.J. (2012). Acid–base crystalline complexes and the pKa rule. CrystEngComm..

[16] Bhogala B.R., Basavoju S., Nangia A. (2005). Tape and layer structures in cocrystals of some di- and tricarboxylic acids with 4,4′-bipyridines and isonicotinamide. From binary to ternary cocrystals. CrystEngComm..

[17] Liu C.-S., Wang J.-J., Yan L.-F., Chang Z., Bu X.-H., Señudo E.C., Ribas J. (2007). Copper(II), Cobalt(II), and Nickel(II) Complexes with a Bulky Anthracene-Based Carboxylic Ligand: Syntheses, Crystal Structures, and Magnetic Properties. Inorg. Chem..

[18] Moulton B., Zaworotko M.J. (2001). From Molecules to Crystal Engineering: Supramolecular Isomerism and Polymorphism in Network Solids. Chem. Rev..

[19] Arici M., Yeşilel O.Z., Acar E., Dege N. (2017). Synthesis, characterization and properties of nicotinamide and isonicotinamide complexes with diverse dicarboxylic acids. Polyhedron.

[20] Moncol J., Mudra M., Lönnecke P., Hewitt M., Valko M., Morris H., Svorec J., Melnik M., Mazur M., Koman M. (2007). Crystal structures and spectroscopic behavior of monomeric, dimeric and polymeric copper(II) chloroacetate adducts with isonicotinamide, N-methylnicotinamide and N,N-diethylnicotinamide. Inorg. Chim. Acta.

[21] Bhogala B.R., Thallapally P.K., Nangia A. (2004). 1:2 and 1:1 Ag(I)-Isonicotinamide coordination compounds: Five-fold interpenetrated CdSO4 network and the first example of (pyridine)N-Ag-O(amide) bonds. Crystal Growth & Design, 4, 215-218. Cryst. Growth Des..

[22] Lynch M.B., Lawrence S.E., Nolan M. (2018). Predicting Nucleation of Isonicotinamide from the Solvent–Solute Interactions of Isonicotinamide in Common Organic Solvents. J. Phys. Chem. A.

[23] Vishweshwar P., Nangia A., Lynch V.M. (2003). Molecular complexes of homologous alkanedicarboxylic acids with isonicotinamide: X-ray crystal structures, hydrogen bond synthons, and melting point alternation. Cryst. Growth Des..

[24] Mohamed S., Tocher D.A., Vickers M., Karamertzanis P.G., Price S.L. (2009). Salt or Cocrystal? A New Series of Crystal Structures Formed from Simple Pyridines and Carboxylic Acids. Cryst. Growth Des..

[25] Sánchez-Férez F., Bayés L., Font-Bardia M., Pons J. (2019). Solvent dependent formation of Cu(II) complexes based on isonicotinamide ligand. Inorg. Chim. Acta.

[26] Soldevila-Sanmartín J., Ayllón J.A., Calvet T., Font-Bardía M., Domingo C., Pons J. (2016). Synthesis, crystal structure and magnetic properties of a Cu(II) paddle-wheel complex with mixed bridges. Inorg. Chem. Commun..

[27] Sánchez-Férez F., Guerrero M., Ayllón J.A., Calvet T., Font-Bardía M., Giner Planas J., Pons J. (2019). Reactivity of homoleptic and heteroleptic core paddle wheel Cu(II) compounds. Inorg. Chim. Acta.

[28] Williams D.H., Fleming I. (1995). Spectroscopic Methods in Organic Chemistry.

[29] Dey A., Kirchner M.T., Vangala V.R., Desiraju G.R., Mondal R., Howard J.A.K. (2005). Crystal Structure Prediction of Aminols: Advantages of a Supramolecular Synthon Approach with Experimental Structures. J. Am. Chem. Soc..

[30] Bavishi D.D., Borkhataria C.H (2016). Spring and parachute: How cocrystals enhance solubility. Prog. Cryst. Growth Charact. Mater..

[31] Shishkin O.V., Zubatyuk R.I., Shishkina S.V., Dyakonenko V.V., Medviediev V.V. (2014). Role of supramolecular synthons in the formation of the supramolecular architecture of molecular crystals revisited from an energetic viewpoint. Phys. Chem. Chem. Phys..

[32] Seo M., Park J., Kim S.Y. (2012). Self-assembly driven by an aromatic primary amide motif. Org. Biomol. Chem..

[33] Tothadi S., Desiraju G.R. (2012). Unusual co-crystal of isonicotinamide: The structural landscape in crystal engineering. Philos. Trans. R. Soc. A.

[34] Nakamoto K. (2009). Infrared and Raman Spectra of Inorganic and Coordination Compounds. Applications in Coordination, Organometallic, and Bioinorganic Chemistry.

[35] Sutton D. (1968). Electronic Spectra of Transition Metal Complexes.

[36] Morse P.M., Girolami G.S. (1989). Are d^0^ ML_6_ Complexes Always Octahedral? The X-ray Structure of Trigonal-Prismatic [Li(tmed)]_2_[ZrMe_6_]. J. Am. Chem. Soc..

[37] Friese J.C., Krol A., Puke C., Kirschbaum K., Giolando D.M. (2000). Trigonal Prismatic vs Octahedral Coordination Geometry: Syntheses and Structural Characterization of Hexakis(arylthiolato) Zirconate Complexes. Inorg. Chem..

[38] Falvello L.R. (1997). Jahn–Teller effects in solid-state co-ordination chemistry. J. Chem. Soc. Dalton Trans..

[39] Nimmermark A., Öhrström L., Reedijk J. (2013). Metal-ligand bond lengths and strengths: Are they correlated? A detailed CSD analysis. Z. Kristallogr..

[40] Adison A.W., Rao T.N. (1984). Synthesis, structure, and spectroscopic properties of copper(II) compounds containing nitrogen–sulphur donor ligands; the crystal and molecular structure of aqua[1,7-bis(N-methylbenzimidazol-2′-yl)-2,6-dithiaheptane]copper(II) perchlorate. J. Chem. Soc. Dalton. Trans..

[41] Gör K., Kürkçüoğlu G.S., Yeşilel O.Z., Büyükgüngör O. (2014). One-dimensional bimetallic cyano complexes with nicotinamide and isonicotinamide ligands. J. Mol. Struct..

[42] Gudavarthy R.V., Burla N., Kulp E.A., Limmer S.J., Sinn E., Switzer J.A. (2011). Epitaxial electrodeposition of chiral CuO films from copper(II) complexes of malic acid on Cu(111) and Cu(110) single crystals. J. Mater. Chem..

[43] Wolff S.K., Grimwood D.J., McKinnon J.J., Jayatilaka D. (2017). and Spackman, M.A. CrystalExplorer17.5.

[44] Évora A.O.L., Bernades C.E.S., Piedade M.F.M., Conceição A.C.L., Minas da Piedade M.E. (2019). Energetics of Glycine Cocrystal or Salt Formation with Two Regioisomers: Fumaric Acid and Maleic Acid. Cryst. Growth Des..

[45] Sheldrick G.M. (2008). A short history of SHELX. Acta Crystallogr. Sect. A Found. Crystallogr..

[46] Macrae C.F., Edgington P.R., McCabe P., Pidcock E., Shields G., Taylor R., Towler M., Van de Streek J.J. (2006). Mercury: Visualization and analysis of crystal structures. Appl. Crystallogr..

[47] Macrae C.F., Bruno I.J., Chisholm J.A., Edgington P.R., McCabe P., Pidcock E., Rodriguez-Monge I., Taylor R., Towler M., van de Streek J. (2008). Mercury CSD 2.0—New features for the visualization and investigation of crystal structures. Appl. Crystallogr..

[48] Persistence of Vision Pty. Ltd. (2004). Persistence of Vision (TM) Raytracer.

