# Supplementary material for: Isonicotinamide-Based Compounds: From Cocrystal to Polymer
Source: Molecules. 2019 Nov 17;24(22):4169. doi: 10.3390/molecules24224169 (PMC6891728; doi:10.3390/molecules24224169)
Supplement: Supplementary file 1 [file molecules-24-04169-s001.pdf]

Supporting Information

Isonicotinamide-Based Compounds: From Cocrystal to Polymer

Francisco Sánchez-Férez [1], Daniel Ejarque [1], Teresa Calvet [2], Mercè Font-Bardia [3] and Josefina Pons [1,*]

[1] Departament de Química, Universitat Autònoma de Barcelona, 08193 Barcelona, Spain; francisco.sanchez.ferez@uab.cat (F.S.-F.); daniel.ejarque@e-campus.uab.cat (D.E.)
[2] Cristal·lografia, Mineralogia i Dipòsits Minerals, Universitat de Barcelona, 08028 Barcelona, Spain; mtcalvet@ub.edu
[3] Unitat de Difracció de Raig-X, Centres Científics i Tecnològics de la Universitat de Barcelona (CCiYUB), Universitat de Barcelona, 08028 Barcelona, Spain; mercef@ccit.ub.edu
* Correspondence: josefina.pons@uab.cat (J.P.); Tel.: +34-935-812-895

FTIR-ATR, ^{1}H NMR and ^{13}C {^{1}H} NMR spectroscopies of compound 2, HPip and Isn

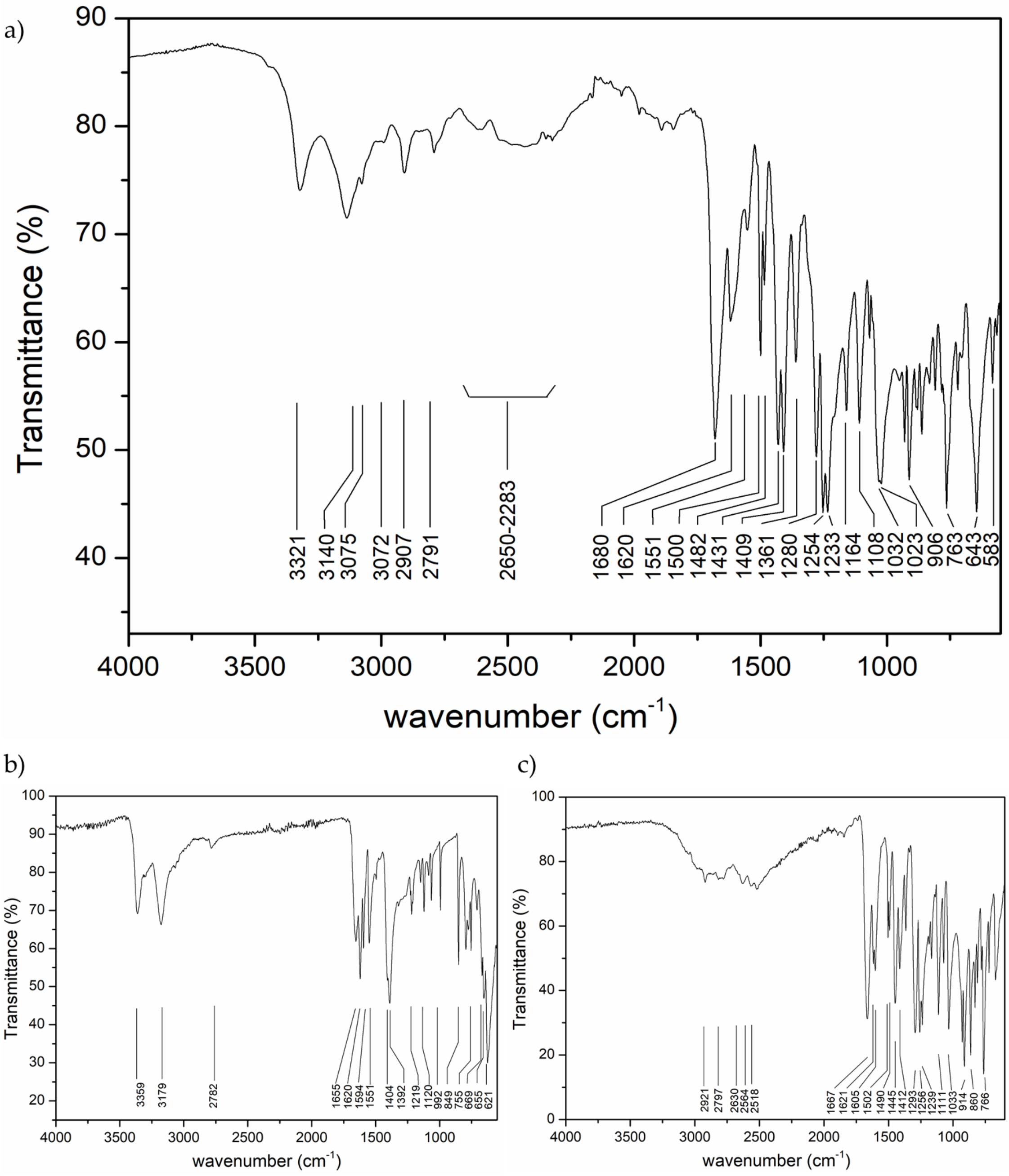

Figure S1. FTIR-ATR spectra of a. compound $(HPip)_2(Isn)$ (**2**); b. Isn and c. HPip.

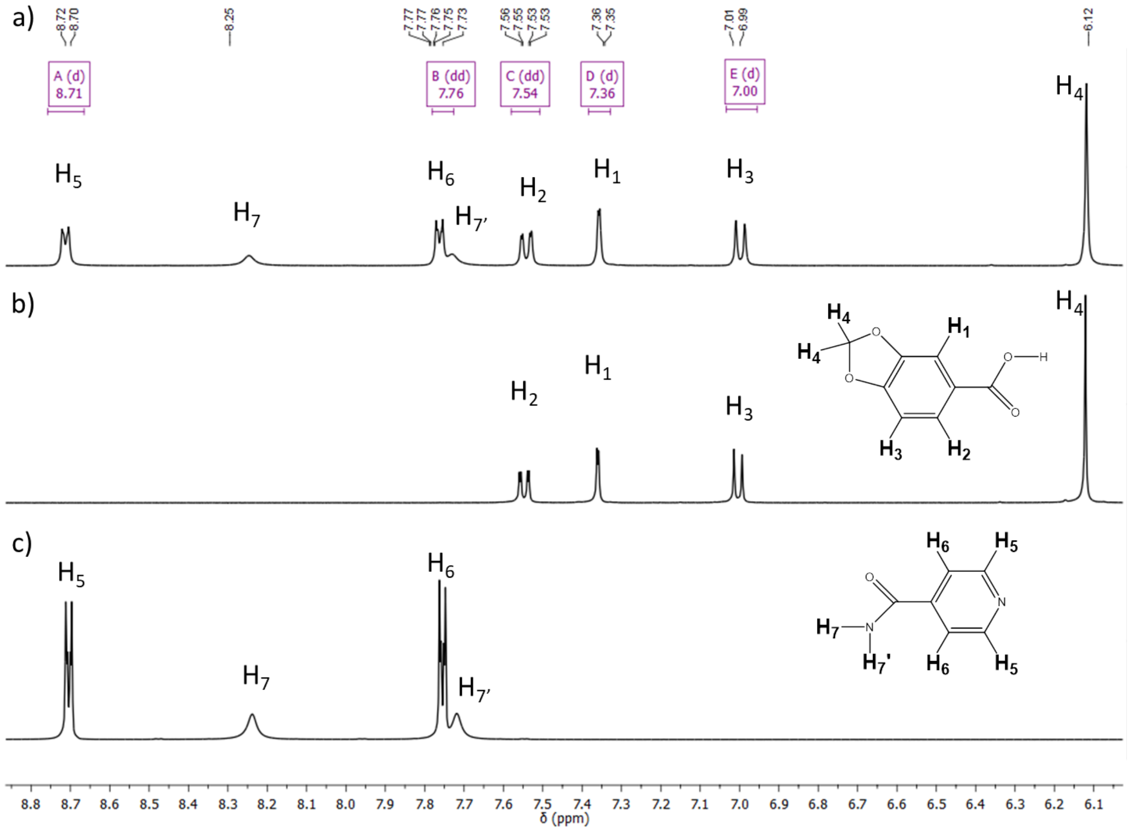

Figure S2. ^{1}H-NMR spectra of a. compound $(HPip)_2(Isn)$ (**2**); b. HPip and c. Isn.

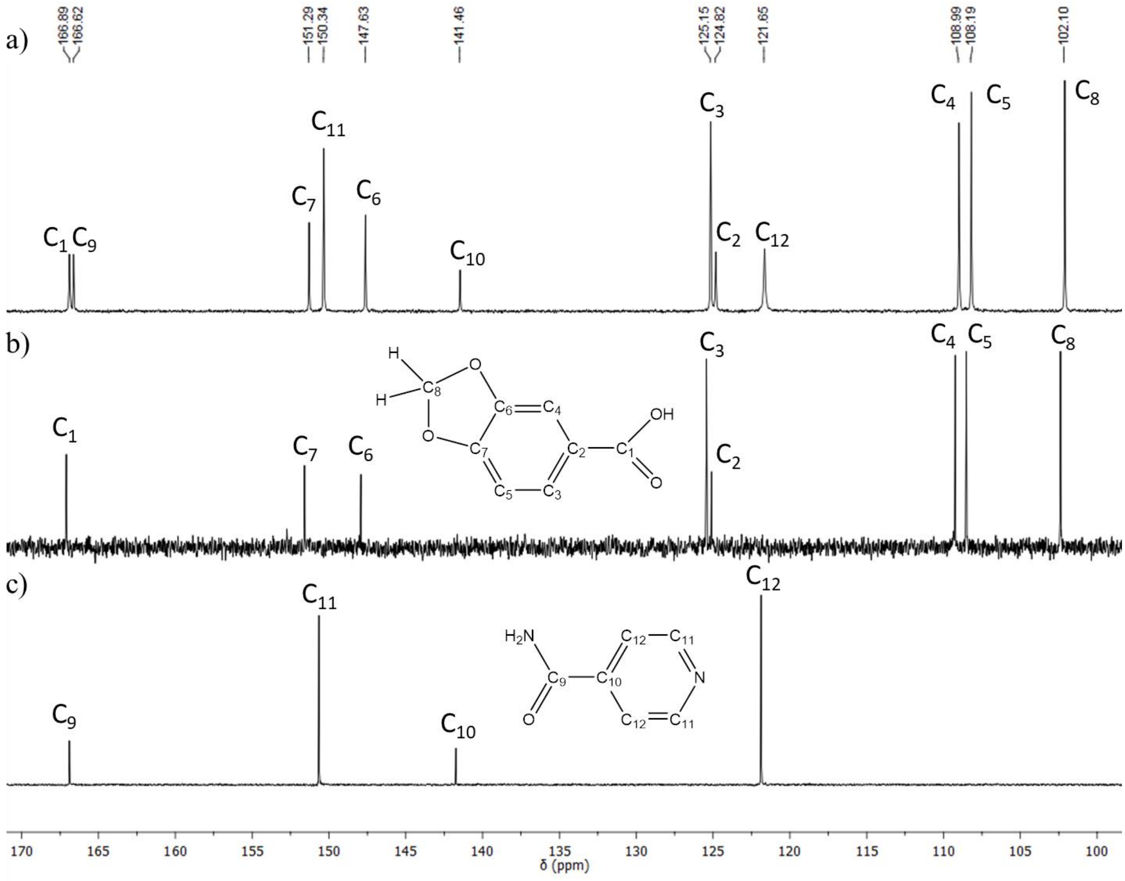

Figure S3. $^{13}C\{^{1}H\}$-NMR spectrum of a. compound $(HPip)_2(Isn)$ (**2**); b**.** HPip and c. Isn.

ATR-FTIR spectroscopy of compounds 3-4

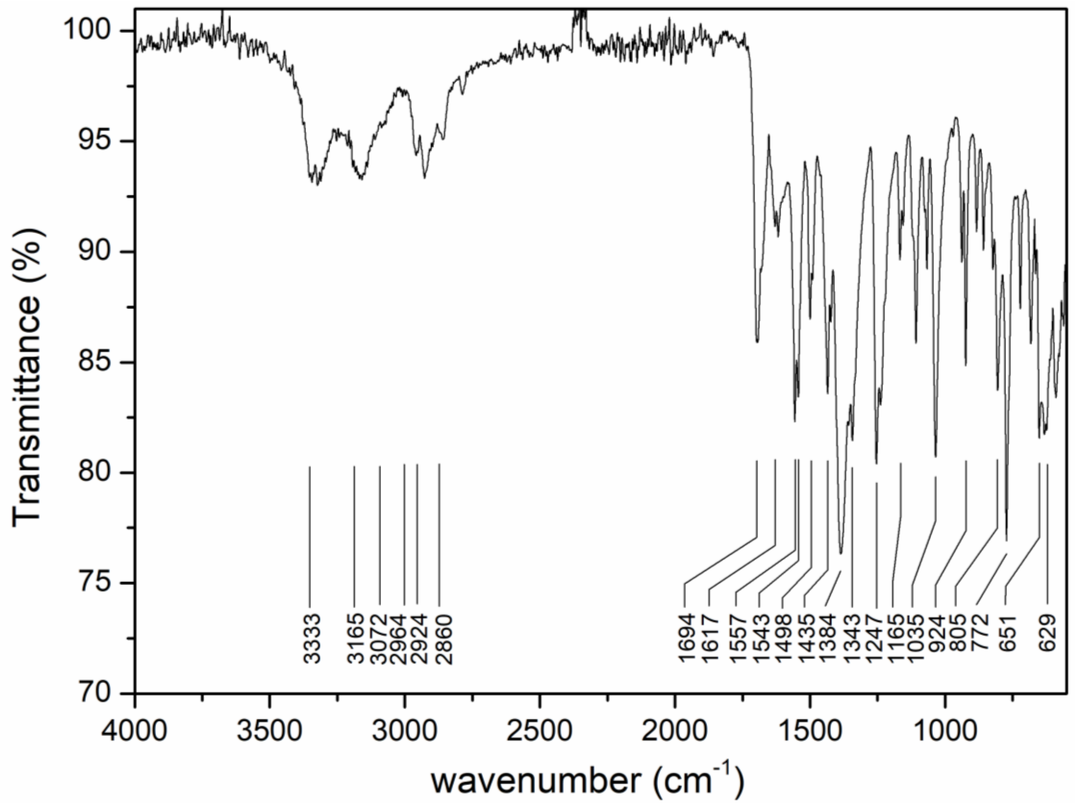

Figure S4. FTIR-ATR spectrum of compound [Cu(Pip)$_2$(Isn)$_2$] (**3**).

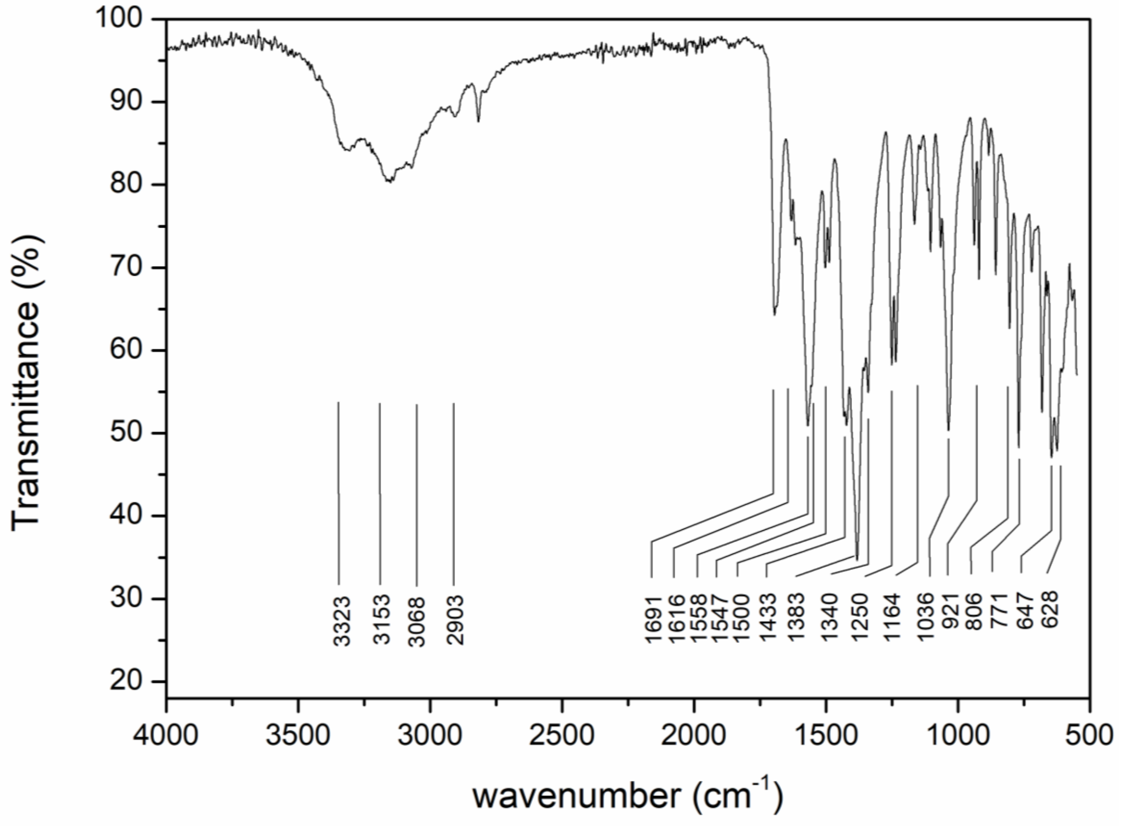

Figure S5. FTIR-ATR spectrum of compound [Cu(Pip)$_2$(Isn)$_2$]·($C_5H_{11}OH$) (**3a**).

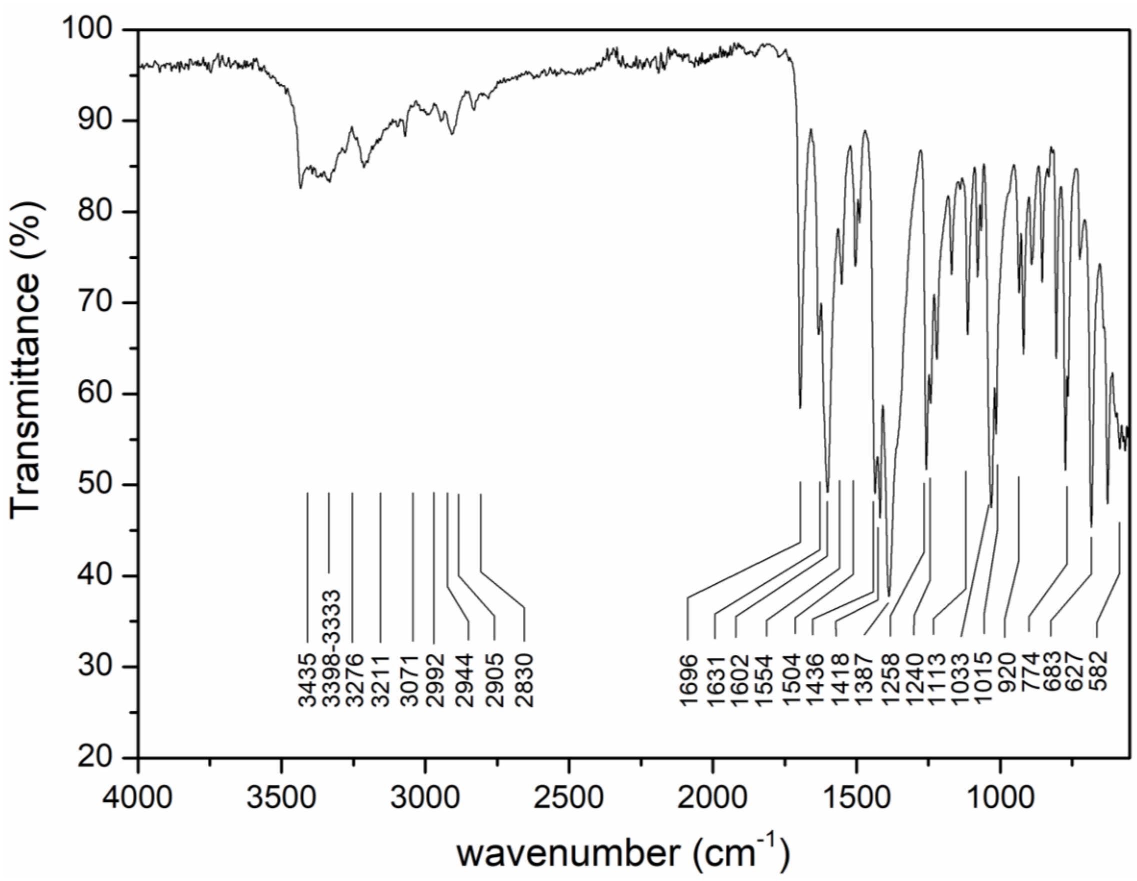

Figure S6. FTIR-ATR spectrum of compound $\{[Cu_3(Pip)_2(OAc)_2(\mu\text{-}Isn)_2(Isn)_2(\mu\text{-}OCH_3)_2(MeOH)_2]\cdot 2MeOH\}_n$ (**4**).

UV-Vis spectroscopy

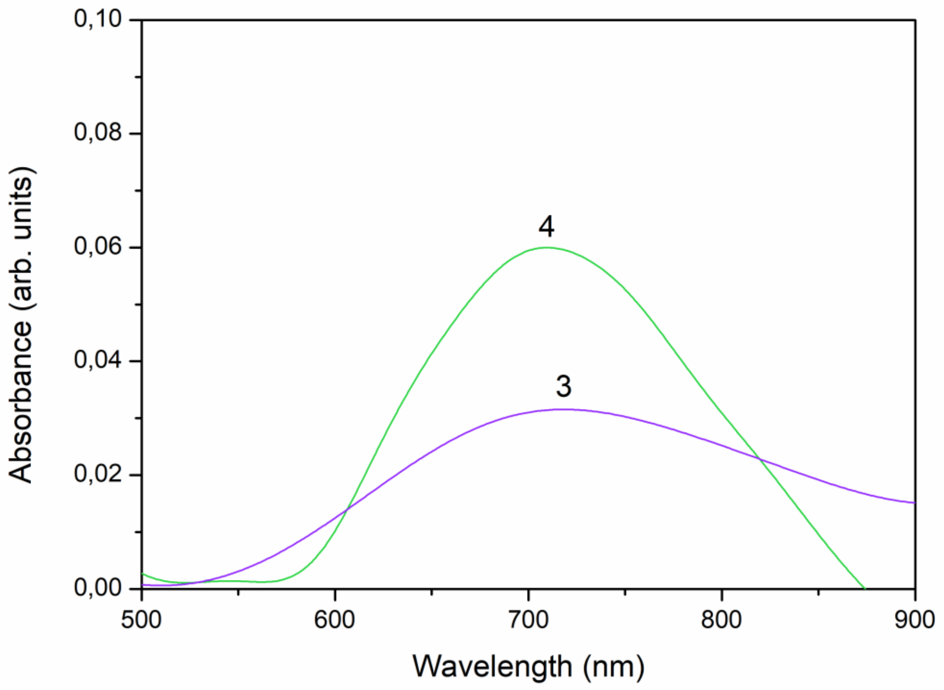

Figure S7. Electronic spectra of compounds **3** and **4** between 500 nm and 900 nm in MeOH solution.

Hirshfeld surfaces analysis

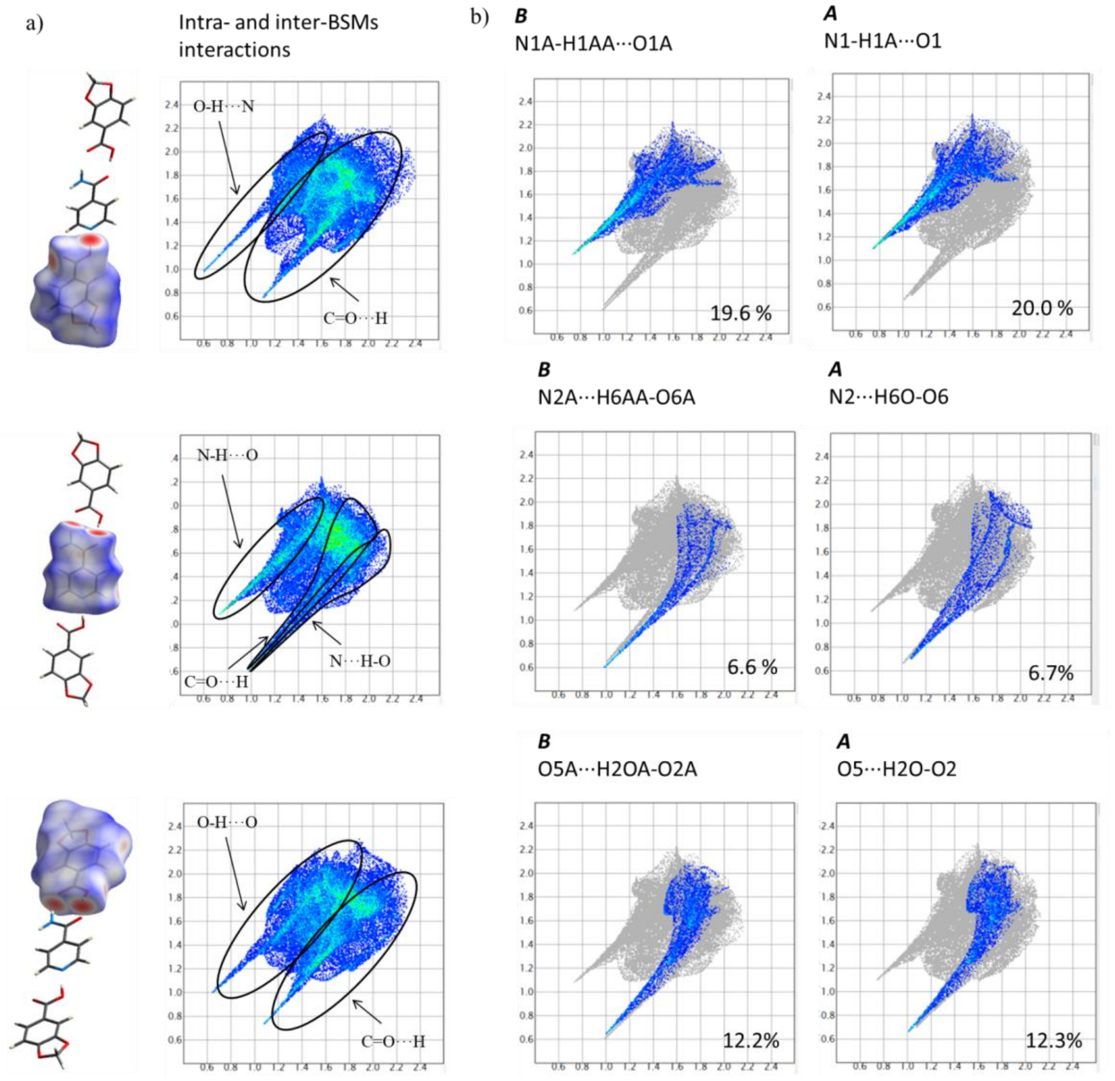

Figure S8. Hirshfeld surface representation and fingerprint plot of **2**. In detail view of a. each molecule which forms the BSMs and b. each intra-BSM interaction with the surface area implied in it.

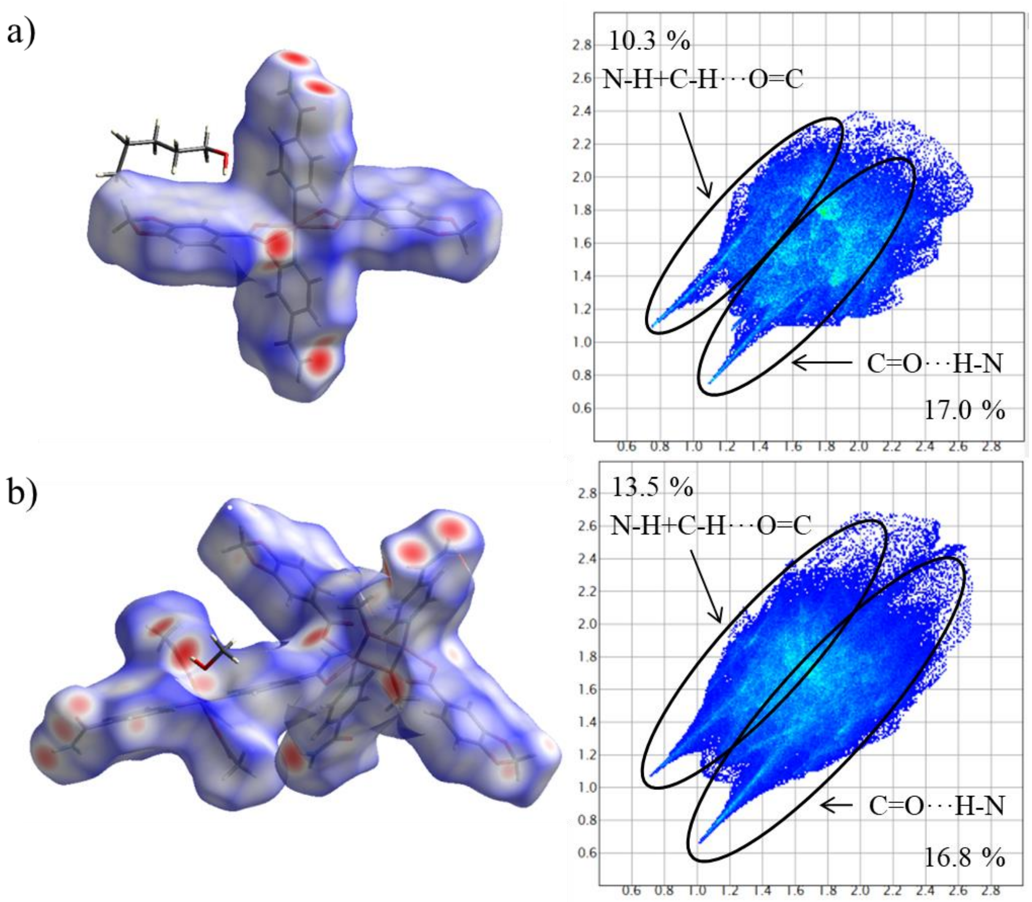

Figure S9. Hirshfeld surface representation and fingerprint plot of a. **3a** and b. **4**.

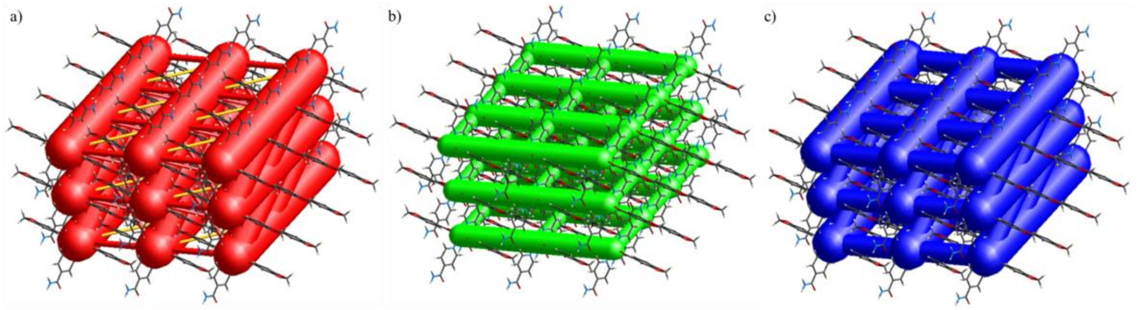

Figure S10. Energy frameworks diagram of a. E_{ele}, b. E_{dis}, c. E_{tot} for compound **3a**. All diagrams use the same energy cylinder scale of 240 and the energy threshold was fixed at 3.0 kJ·mol^{-1} for E_{ele} and 30.0 kJ·mol^{-1} for E_{dis} and E_{tot}. Color codes of energy frameworks are red (E_{ele}); green (E_{dis}); blue (E_{tot}) and yellow (destabilizing energies).
